# Prediction of leukocyte counts during paediatric acute lymphoblastic leukaemia maintenance therapy

**DOI:** 10.1038/s41598-019-54492-5

**Published:** 2019-12-02

**Authors:** Santeri Karppinen, Olli Lohi, Matti Vihola

**Affiliations:** 1 University of Jyväskylä, Department of Mathematics and Statistics, Jyväskylä, FI-40014 Finland; 2 Tampere Center for Child Health Research, Faculty of Medicine and Health Technology, Tampere University and Tampere University Hospital, Tampere, FI-33521 Finland

**Keywords:** Stochastic modelling, Time series, Paediatric cancer, Applied mathematics, Statistics

## Abstract

Maintenance chemotherapy with oral 6-mercaptopurine and methotrexate remains a cornerstone of modern therapy for acute lymphoblastic leukaemia. The dosage and intensity of therapy are based on surrogate markers such as peripheral blood leukocyte and neutrophil counts. Dosage based leukocyte count predictions could provide support for dosage decisions clinicians face trying to find and maintain an appropriate dosage for the individual patient. We present two Bayesian nonlinear state space models for predicting patient leukocyte counts during the maintenance therapy. The models simplify some aspects of previously proposed models but allow for some extra flexibility. Our second model is an extension which accounts for extra variation in the leukocyte count due to a treatment adversity, infections, using C-reactive protein as a surrogate. The predictive performances of our models are compared against a model from the literature using time series cross-validation with patient data. In our experiments, our simplified models appear more robust and deliver competitive results with the model from the literature.

## Introduction

Acute lymphoblastic leukaemia (ALL) is the most common cancer in childhood. In the Nordic countries, approximately 210 children are diagnosed yearly and patients are treated with chemotherapeutic drugs according to the ALL protocols of the Nordic Society of Paediatric Haematology and Oncology (NOPHO)^1^. The last phase of the treatment, maintenance therapy (MT), continues until 2 to 3 years from diagnosis. During MT, patients are treated orally with daily 6-mercaptopurine (6 MP) and weekly methotrexate (MTX).

Conventional MT starts with a standard 6 MP/MTX dose defined in the protocol. After initialisation of treatment, the dosage of the cytotoxic drugs is adjusted to reach a degree of myelosuppression, reflected in the NOPHO ALL-2008 protocol by targeting a leukocyte count of 1.5–3.0 × 10^9^/L, while keeping the neutrophil count above 0.5 × 10^9^/L^2^. Individual adjustments of 6 MP/MTX doses are necessary due to substantial interindividual variability in 6 MP/MTX bioavailability and cellular pharmacokinetics, and a narrow therapeutic index.

Finding the right 6 MP/MTX dosage may be challenging because there is a substantial delay before steady-state response in the leukocyte count is reached. Furthermore, many other factors, such as infections, can cause leukocyte fluctuations, and the dosage decisions during MT may be made by clinicians who have limited prior experience with 6 MP/MTX chemotherapy. Making the right decisions is crucial, as excessive dosage is associated with acute toxicity^3^ and the risk of second cancers^4^, whereas insufficient dosage results in poor treatment outcomes^5–7^.

In this work, we develop statistical models for predicting leukocyte counts based on the doses administered during MT. One motivation for our work is a potential future application, where predictive modelling would be a part of a dosage decision support system, which automatically fits the model with data accumulated for the patient so far. The system then provides the clinician with an interactive visualisation of the patient’s data, and leukocyte count predictions under alternative future dosing scenarios. This offers the clinician an analytical look on the data, and reassurance on her dosage decision. Ideally, the system could provide reliable predictions for most of the patients, but the clinician’s expertise would remain essential for decision-making under exceptional scenarios such as patients with rare genotypes that affect 6 MP metabolism or patients with an infection.

The scope of this work is in the development of the predictive models, and in the evaluation of their predictive accuracies. We do not consider the implementation of the models into the clinical practice, or suggest alternative dosing strategies. We focus on the mathematical modelling related to the prediction of leukocyte counts in the context of ALL, but our developments may also be relevant outside this context, for instance in computational personalised medicine regarding other myelosuppressive medication. Currently, there are two published works where leukocyte counts during ALL MT are predicted^8,9^. Here, we present two statistical models following a structure similar to the existing models, but instead of using ordinary differential equation models, we use nonlinear Gaussian state space models^10^ that stem from analogous stochastic differential equations. Our models introduce two simplifications, on the pharmacokinetic model for 6 MP^11^ and on the leukopoiesis model^8,12^. Our second model, an extension of the first, incorporates C-reactive protein (CRP) measurements as a surrogate for infections and models the effect of an infection as extra variation in (or discrepancy from) the leukopoiesis model.

## Methods

The patient data were collected from historical medical records and consist of 23 patients under the age of 18 who had received MT under the NOPHO ALL-2000 or ALL-2008 treatment protocols at the Tampere University Hospital in Finland. This registry study (R16527) was accepted by the director of the Science Center in the Tampere University Hospital according to the local practice, and the data were anonymized before further analysis. The treatment length per patient varies from 227 to 524 days, with most of the patients receiving MT for more than 400 days. For each patient, the data contain the daily 6 MP dosage prescribed, as well as the leukocyte count and the CRP measurements made typically during weekly or biweekly visits to the hospital or the laboratory. The height and weight of each patient is also available at the start of MT. We used the Mosteller formula^13^ to calculate the body surface area (BSA) for all patients during the treatment. The height and weight gain of the patients during the treatment was estimated by interpolating median growth curves obtained from the Centers for Disease Control and Prevention^14^. Because the patients’ genders are not available in the data, average growth curves over boys and girls aged under 20 years were used. For each patient, the interpolation was begun from the height and weight values recorded in the data. The patientwise time series of the leukocyte counts, 6 MP, CRP and BSA are in the Supplementary Dataset 1.

To compare the models, we use the root mean squared error (RMSE) and the mean absolute error (MAE). In addition, we compute *α*% coverage probabilities, that is, ${{\rm{CP}}}_{\alpha }=\frac{1}{n}\,{\sum }_{i=1}^{n}\,{\bf{1}}\{{y}_{i}\in {{\rm{I}}}_{i}^{\alpha }\}$ for $\alpha \in \{50,90\}$, where *y*_*i*_ denotes observation number *i* and ${{\rm{I}}}_{i}^{\alpha }$ denotes the *α*% probability interval for observation *y*_*i*_. This metric is used to evaluate the ability of the models to quantify the uncertainty related to the point predictions. All of the metrics are computed out-of-sample and in-sample.

The out-of-sample metrics are of most interest, as they are computed using data not used in the model fitting and are directly tied to the predictive performance of the models. In a time series context, a natural way to compute them is to use time series cross-validation^15^ (TSC). In a single round of TSC, we partition the data to a training set with data up to time $t$, and a prediction horizon immediately following the training set. The model is fit using the training set and the observations that fall into the prediction horizon are predicted using the fitted model. The training dataset is then augmented with observations in the prediction horizon and the process repeats until the data have been exhausted. After TSC, we compute the metrics using the obtained predictions and the corresponding observations. In the in-sample case, the metrics are computed based on model fits to full datasets by predicting all of the observations that were also used in the model fitting.

In the following subsections, we discuss the predictive models and estimation methods. We denote model state variables with capital letters, and parameters and data values in lowercase. A glossary and details regarding symbols used in the model definitions are also given in the Supplementary Tables 1–5.

### Jayachandran *et al*. model (JM)

The model from the literature, which we refer to as JM, is a joint 8-compartment model based on the work of Jayachandran *et al*.^8,11^ The model consists of two submodels, the first of which is the 3-compartment pharmacokinetic model^11^ for the metabolisation of 6 MP to red blood cell 6-thioguanine (TGNRBC):

1 $$\begin{array}{rcl}{\rm{d}}{X}_{gut}/{\rm{d}}t & = & -\,{k}_{ab}{X}_{gut}+d(t)\\ {\rm{d}}{X}_{plasma}/{\rm{d}}t & = & {k}_{ab}{X}_{gut}-{k}_{el}{X}_{plasma}-\frac{{k}_{cm}{X}_{plasma}}{k+{X}_{plasma}}\\ {\rm{d}}{X}_{tgn}/{\rm{d}}t & = & \frac{{v}_{cm}{k}_{cm}{X}_{plasma}}{k+{X}_{plasma}}-{k}_{me}{X}_{tgn}.\end{array}$$

The authors assume TGNRBC to be associated with the toxicity in the bone marrow due to 6 MP and hence model the variable as a surrogate for the myelosuppressive effect of 6 MP. The dataset in the article contained the administered 6 MP doses and the measured TGNRBC concentrations. The compartments ${X}_{gut}$ and ${X}_{plasma}$ represent 6 MP in gut and plasma, ${X}_{tgn}$ is the TGNRBC compartment, $d(t)$ is the dose input at time *t* and the remaining symbols are parameters. The functional form of $d(t)$ was not given^8,11^. Hence, we assume $d(t)$ equals zero unless a dose is given exactly at time *t*.

The second submodel is the leukopoiesis model by Jayachandran *et al*.^8^, which is a modification of the widely used 5-compartment model introduced by Friberg *et al*.^12^. We detail the model for log-transformed state variables:

2 $$\begin{array}{rcl}{\rm{d}}S/{\rm{d}}t & = & {k}_{pl}^{max}\frac{{\rho }^{\gamma }}{{\rho }^{\gamma }+\exp {(L)}^{\gamma }}-\frac{{e}_{max}{X}_{tgn}}{{e}_{C50}+{X}_{tgn}}-{k}_{tr}\\ {\rm{d}}{C}^{(1)}/{\rm{d}}t & = & {k}_{tr}\,\exp (S-{C}^{(1)})-{k}_{tr}\\ {\rm{d}}{C}^{(2)}/{\rm{d}}t & = & {k}_{tr}\,\exp ({C}^{(1)}-{C}^{(2)})-{k}_{tr}\\ {\rm{d}}{C}^{(3)}/{\rm{d}}t & = & {k}_{tr}\,\exp ({C}^{(2)}-{C}^{(3)})-{k}_{tr}\\ {\rm{d}}L/{\rm{d}}t & = & {k}_{tr}\,\exp ({C}^{(3)}-L)-{k}_{L}.\end{array}$$

Here, the state variables form a maturation chain from stem cells (*S*) to leukocytes in circulation (*L*) through three maturation phases denoted by the compartments ${C}^{(i)},i=1,2,3$. TGNRBC (${X}_{tgn}$) is assumed to diminish the rate of stem cell production. The remaining symbols are parameters.

As no information regarding the initial values of (1) or (2) is given^8,11^, we assume that the patient’s system starts in a steady state where no change in the cell concentrations is occuring initially. The steady state initialisation is obtained by setting the time derivatives at the start of the treatment (time zero) to zero. This is achieved by:

3 $$\begin{array}{rcl}L(0) & = & \log (\rho )+\,\log (\frac{{k}_{pl}^{max}}{{k}_{tr}}-1)/\gamma \\ {C}^{(3)}(0) & = & \log ({k}_{L})-\,\log ({k}_{tr})+L(0)\\ S(0) & = & {C}^{(1)}(0)={C}^{(2)}(0)={C}^{(3)}(0),\end{array}$$

whenever ${k}_{tr} < {k}_{pl}^{max}$. Furthermore, we assume no 6 MP or TGNRBC exists in the patient’s system at the beginning of MT, i.e. ${X}_{gut}(0)={X}_{plasma}(0)={X}_{tgn}(0)=0$.

The log-leukocyte count measurements of a patient, ${({l}_{k})}_{k\ge 1}$, observed at times *t*_*k*_, are assumed i.i.d. with Gaussian errors:

4 $${l}_{k}\sim N(\hat{L}({t}_{k},\theta ,{d}_{1:k}),{\sigma }_{leuk}^{2}),$$

where $\hat{L}({t}_{k},\theta ,{d}_{1:k})$ is the solution of the state variable *L* at time *t*_*k*_, dependent on patient specific parameters *θ* and administered doses up to time index *k*, ${d}_{1:k}$.

### 2-compartment model (TCM)

Our first model, denoted TCM, can be seen as a K-PD model^16^. TCM has a structure similar to that of JM, which it simplifies in two ways.

First, the pharmacokinetic model (1) is replaced with the pharmacokinetic model

5 $$\frac{{\rm{d}}M(t)}{{\rm{d}}t}={k}_{me}\frac{{e}_{tgn}d(t)}{d(t)+h}-{k}_{me}M(t).$$

The model reflects changes in the cytotoxicity induced by 6 MP, *M*, in response to the 6 MP dose administered to the patient. The value of *M* models the direct effect of chemotherapy, and is the counterpart of the term $\frac{{e}_{max}{X}_{tgn}}{{e}_{C50}+{X}_{tgn}}$ in (2). The value of the drug input function at time *t*, $d(t)$, equals the last 6 MP dose administered during the last 24 (${T}_{dur}$) hours normalised by the patient’s BSA, and zero if no dose was given. While this leads to noticeably different behaviour compared to (1) in the hourly time scale, the average daily behaviour of $M(t)$ remains very similar to that of ${X}_{tgn}$. A similar observation is made by Le *et al*.^9^, who note that varying ${T}_{dur}$ does not have a strong influence on the concentration of TGNRBC in a prior pharmacokinetic model introduced by Jayachandran *et al*.^8^, which is very similar to (1). Like the pharmacokinetic model of JM, (5) concentrates on the cytotoxic effect of 6 MP, and does not include MTX. We return to this matter in the discussion.

The parameters ${e}_{tgn}$ and $h$ play roles similar to ${k}_{cm}$ and $k$ in (1) as is evident from the similar functional form of (5) and the differential equation for ${X}_{tgn}$. Furthermore, the parameter ${k}_{me}$ is equivalent in (1) and (5). Jayachandran *et al*. reported a very high posterior correlation between the parameters ${k}_{me}$ and ${k}_{cm}$ in (1) ^11^. We incorporate ${k}_{me}$ into the first term of (5) as this reduces the correlation between ${k}_{me}$ and ${e}_{tgn}$. The simplified form of (5) is motivated by simulation and parameter estimation, which reveal that the functional form of (5) is flexible enough to match solutions of ${X}_{tgn}$ when most of the parameters in (1) are fixed as in the analysis of Jayachandran *et al*.

The second simplification concerns the leukopoiesis model (2), which is replaced with a stochastic differential equation analogue of the equation for $S$:

6 $$d{L}_{t}=({k}_{pl}^{max}\frac{{\rho }^{\gamma }}{{\rho }^{\gamma }+\exp {(L)}^{\gamma }}-{M}_{t}-{k}_{L})dt+{\sigma }_{L}d{B}_{t}^{(L)},$$

where ${B}_{t}^{(L)}$ is the Brownian motion and the parameter *σ*_*L*_ is the leukopoiesis standard deviation. The parameter ${k}_{tr}$ in the equation for *S* is substituted by the leukocyte elimination rate ${k}_{L}$ in (6), as (6) is a model for leukocyte counts. The leukopoiesis model (6) eliminates the cell maturation chain in (2) and models the effect of chemotherapy directly on the leukocytes in circulation. Unlike in (2), the drug effect is linear.

To obtain the state equation of TCM, (7), we solve the piecewise linear differential Eq. (5) at each interval $$[{t}_{k-1},{t}_{k})$$ with the initial condition $$M({t}_{k-1})={M}_{k-1}$$, and apply the Euler-Maruyama discretisation^17^ to (6), which results in

7 $$\begin{array}{rcl}{M}_{k} & = & ({M}_{k-1}-\frac{{e}_{tgn}{d}_{k-1}^{BSA}}{{d}_{k-1}^{BSA}+h})\,\exp (\,-\,{k}_{me}\Delta {t}_{k})+\frac{{e}_{tgn}{d}_{k-1}^{BSA}}{{d}_{k-1}^{BSA}+h}\\ {L}_{k} & = & {L}_{k-1}+\Delta {t}_{k}({k}_{pl}^{max}\frac{{\rho }^{\gamma }}{{\rho }^{\gamma }+\exp {({L}_{k-1})}^{\gamma }}-{M}_{k-1}-{k}_{L})+{\sigma }_{L}\sqrt{\Delta {t}_{k}}{\zeta }_{k},\end{array}$$

where ${d}_{k-1}^{BSA}=d({t}_{k-1})$, $\Delta {t}_{k}={t}_{k}-{t}_{k-1}$, and ${\zeta }_{k}$ are standard normal random variables for all *k*. Initial distributions ${M}_{1}\sim N(0,0)$ and ${L}_{1}\sim N({l}_{1},0.5)$ are assumed for the state variables.

The log-leukocyte counts are related to the state variable *L* with the observation equation

8 $${l}_{k}={L}_{k}+{\varepsilon }_{k}^{leuk},\,{\varepsilon }_{k}^{leuk}\sim N(0,{\sigma }_{leuk}^{2}).$$

### 2-compartment model with incorporated CRP (TCM-CRP)

Our second model, denoted TCM-CRP, is an extension of TCM, where the leukopoiesis standard deviation *σ*_*L*_ is inflated in case of infection, for which the patient CRP measurements are taken as a surrogate.

TCM-CRP appends the state Eq. (7) with a third equation concerning an additional state variable, *V*, the level of infection. We model *V* using an Ornstein-Uhlenbeck process:

9 $$d{V}_{t}=[{\theta }_{ou}{V}_{t}]dt+{\sigma }_{ou}d{B}_{t}^{(V)},\,{V}_{0}={v}_{0},$$

where *t* denotes time, ${B}_{t}^{(V)}$ is the Brownian motion, and ${\theta }_{ou}$ and ${\sigma }_{ou}$ are parameters. Conditional on the previous value in the series, *V* in (9) is Gaussian^18^, which leads to the following state equation:

10 $${V}_{k}={V}_{k-1}{e}^{-{\theta }_{ou}\Delta {t}_{k}}+{\sigma }_{ou}{(2{\theta }_{ou})}^{-1/2}{e}^{-{\theta }_{ou}\Delta {t}_{k}}\sqrt{{e}^{2{\theta }_{ou}\Delta {t}_{k}}-1}{\eta }_{k},$$

where *k* denotes the index of the time point and ${\eta }_{k}$ are standard normal random variables for all *k*. The only modification to (7) in TCM-CRP is that ${\sigma }_{L}$ is set to depend on ${V}_{k}$ and parameters ${\sigma }_{L}^{0}$ and ${\beta }_{crp}$ by

11 $${\sigma }_{L}({V}_{k})={\sigma }_{L}^{0}\,\exp ({\beta }_{crp}{V}_{k}),$$

making TCM-CRP a stochastic volatility type model. The state equation for TCM-CRP then consists of (7) modified with (11), and (10). The distribution of *V*_1_ is set to the stationary distribution of (9),

$$N(0,{(\frac{{\sigma }_{ou}}{\sqrt{2{\theta }_{ou}}})}^{2}),$$

and the distributions for *M*_1_ and *L*_1_ remain as in TCM.

Finally, TCM-CRP incorporates the $\log (x+1)$-transformed CRP measurements, *v*_*k*_, into the observation Eq. (8) by setting

12 $${v}_{k}={V}_{k}+{\varepsilon }_{k}^{crp},\,{\varepsilon }_{k}^{crp}\sim N(0,{\sigma }_{crp}^{2}).$$

### Naive mean model (NM)

The fourth model we consider is a naive mean model (NM), which assumes that the leukocyte counts are i.i.d. and follow the normal distribution $N({\mu }_{nm},{\sigma }_{nm}^{2})$. This model is an oversimplification, as it does not take into account the dosage given to the patient. Hence, we consider NM as a baseline for the models TCM, TCM-CRP and JM, and not as a realistic model candidate for predicting leukocyte counts.

### Estimation methods

To estimate the parameters of the models TCM, TCM-CRP and JM, we use maximum a posteriori (MAP) estimation, where the posterior density

13 $$p(\theta |y)\propto p(y|\theta )p(\theta ),$$

is maximised with respect to the logarithm of the free parameters, *θ*, in the model. In (13), *y* denotes the dataset for a single patient.

The value of $p(y|\theta )$ in (13) for JM and a given *θ* stems from (4). We use the Rosenbrock23 method^19^ of the DifferentialEquations.jl package^20^ in the Julia programming language^21^ to solve the systems of differential equations. The predictions for JM are obtained by estimating the free parameters with data up to time index *k*, ${y}_{1:k}$, and solving the resulting system of differential equations on the interval $[{t}_{k+1},{t}_{k+{h}_{pred}}]$, where ${h}_{pred}$ denotes the length of the prediction horizon.

To compute $p(y|\theta )$ and the predictions for TCM and TCM-CRP, we use the extended Kalman filter (EKF) which is an approximate method for computing the filtered state distributions for state space models with nonlinear dynamics in the state and observation equations^10,22^.

In all maximisation problems, we assume the joint prior distribution $p(\theta )$ in (13) consists of vague independent $N(0,10)$ distributions for each free parameter. The Nelder-Mead method^23,24^ in the Optim.jl package^25^ is used for the computation.

To estimate the parameters of the model NM, we compute the sample mean and variance of the leukocyte counts.

## Results

With JM, we attempted to reproduce the analysis of Jayachandran *et al*.^8,11^ as accurately as possible and hence estimated parameters ${k}_{cm}$ in (1) and ${k}_{tr}$, ${k}_{pl}^{max}$, ${k}_{L}$, $\gamma$ and ${e}_{max}$ in (2). These parameters were found to have the greatest influence on the fitted values of JM’s submodels in sensitivity analyses conducted in both articles^8,11^. In addition, the parameter ${\sigma }_{leuk}$ was estimated. The remaining parameters were fixed to the values reported by Jayachandran *et al*.

With TCM, the parameters ${e}_{tgn}$, *h*, ${k}_{pl}^{max}$, ${k}_{L}$ and *σ*_*L*_ were estimated. The common parameters with JM, ${k}_{pl}^{max}$ and ${k}_{L}$, were estimated, but we fixed *γ* to a value reported by Jayachandran *et al*.^8^, because estimating it resulted in fits with oscillating behaviour not visible in the datasets. Furthermore, we estimated the leukopoiesis standard deviation ${\sigma }_{L}$, but fixed the measurement standard deviation ${\sigma }_{leuk}$ to a literature value of 0.057 for the accuracy of measuring neutrophil counts^26^. The remaining parameters, ${k}_{me}$ and $\rho$, were fixed to the same values as in JM. The discretisation $\Delta {t}_{k}$ was set to 0.25.

TCM-CRP was treated similarly to TCM, with the parameter ${\sigma }_{L}^{0}$ as the equivalent of ${\sigma }_{L}$. However, to maintain the same amount of free parameters as in TCM, we fixed the additional parameters ${\sigma }_{crp}$, ${\sigma }_{ou}$, ${\theta }_{ou}$ and ${\beta }_{crp}$. As the coefficient of variation for measuring CRP at 3.5 mg/l is close to 10%^27^ and $\log (x+1)\approx \,\log (x)$ when $x\ge 3.5$, we fixed ${\sigma }_{crp}=0.1$ (note that if $X\sim N(\mu ,{\sigma }^{2})$, $\mu  > 0$ and *σ*^2^ sufficiently small, then $\log (X)\sim N(\log (\mu ),{(\sigma /\mu )}^{2})$ approximately). The remaining parameters, ${\theta }_{V}=({\sigma }_{ou},{\theta }_{ou},{\beta }_{crp})$, were fixed to estimates obtained by maximising the objective

14 $$\prod _{i}\,p({y}_{i}|{\theta }_{i},{\theta }_{V})p({\theta }_{i},{\theta }_{V})$$

with respect to $({\theta }_{1},{\theta }_{2},\ldots ,{\theta }_{23},{\theta }_{V})$. In (14) each patient is indexed with *i*; ${y}_{i}$ and ${\theta }_{i}$ denote the dataset and the parameter vector of the free parameters in TCM for patient *i*. The joint approach for obtaining an estimate of ${\theta }_{V}$ was motivated by the fact that if ${\theta }_{V}$ were estimated individually for each patient, inadequate estimates of ${\beta }_{crp}$ were obtained for patients with mild or no infections during their treatment.

For all models, TSC was carried out such that the first training dataset for each patient was set to contain the first 8 weeks of the patient’s data. In one case however, the first 8 weeks contained only one measured leukocyte count, and hence the first training set was extended to include two observations. For all models, TSC was run twice, with a prediction horizon of two and four weeks. The TSC schemes were completed successfully for the models TCM and TCM-CRP. With the two and four week schemes of JM, there were 31 and 19 TSC rounds where optimisation did not converge or prediction failed with a solver error. The patients who had at least one convergence or prediction failure during TSC with any horizon were 1, 4, 6, 7, 8, 11, 12, 20 and 22. Furthermore, when the models were fit to the full datasets, the optimisation of the parameters of JM did not converge for patient 6. In the summary tables that follow, the problematic TSC rounds and fits have been removed prior to computing the metrics. In the patientwise listings, these have not been removed.

The out-of-sample metrics with both of the prediction horizons are given in Table 1. The tabulated values are means over the metrics computed for each patient (underlying data available in the Supplementary Dataset 2). To compute the values, the logarithmic scale predictions of the models TCM, TCM-CRP and JM have been transformed to the linear scale. In the table, the means of RMSE and MAE suggest that the point predictive accuracies of TCM and TCM-CRP are slightly greater than the predictive accuracy of JM regardless of the prediction horizon. The baseline model NM performs surprisingly well and is roughly as accurate as JM.

**Table 1:** The out-of-sample metrics for the models (TCM, TCM-CRP and JM) and the baseline model NM with both of the prediction horizons: means of coverage probability (CP), mean absolute error (MAE) and root mean squared error (RMSE).

	2 weeks				4 weeks			
	TCM	TCM-CRP	JM	NM	TCM	TCM-CRP	JM	NM
${\overline{{\rm{CP}}}}_{50}$	0.457 (0.13)	0.442 (0.13)	0.421 (0.13)	0.514 (0.15)	0.443 (0.15)	0.428 (0.14)	0.402 (0.15)	0.507 (0.15)
${\overline{{\rm{CP}}}}_{90}$	0.795 (0.11)	0.787 (0.11)	0.761 (0.14)	0.873 (0.09)	0.767 (0.13)	0.759 (0.13)	0.722 (0.17)	0.863 (0.09)
$\overline{{\rm{MAE}}}$	0.860 (0.33)	0.870 (0.35)	0.964 (0.42)	0.986 (0.43)	0.896 (0.34)	0.914 (0.38)	1.016 (0.45)	1.001 (0.44)
$\overline{{\rm{RMSE}}}$	1.232 (0.51)	1.244 (0.53)	1.387 (0.66)	1.308 (0.57)	1.278 (0.55)	1.309 (0.64)	1.430 (0.68)	1.326 (0.59)

Standard deviations are in parentheses. Similar means are obtained if the metrics are computed modelwise without considering the patients separately.

The widths of the predictive probability intervals are closer to their target values for TCM and TCM-CRP than JM: in the case of the two week horizon, we observe discrepancies of 4–6% vs. 8% for CP_50_ and discrepancies of 10–11% vs. 14% for CP_90_. The respective discrepancies increase to about 6–7% vs. 10% for CP_50_ and 13–14% vs. 18% for CP_90_, when the horizon is extended to four weeks. The models TCM, TCM-CRP and JM underestimate the width of the intervals. This is likely a consequence of using MAP estimation, which does not account for the uncertainty in the model parameters. A more accurate representation of the uncertainty in the predictions could be obtained for example by using Markov chain Monte Carlo methods^28^ that produce samples from the full posterior.

**Figure 1:**
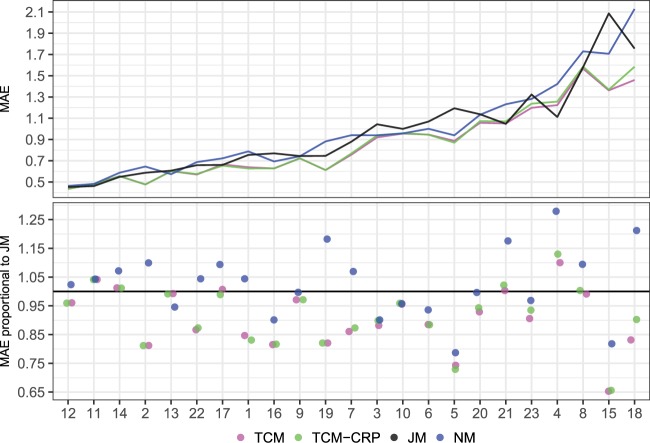
The patientwise out-of-sample mean absolute error for the models (TCM, TCM-CRP, JM) and the baseline model NM (top), and the relative error with respect to JM (bottom). The black line in the bottom plot depicts the line of equal predictive accuracy with JM. The out-of-sample values are from time series cross-validation with the two week prediction horizon. Each model is represented by a color. The patients have been ordered with increasing mean MAE over the models.

The predictive metrics are examined further in Fig. 1, which plots the patientwise MAE of the models in case of the two week prediction horizon. The plot shows that for most of the patients, TCM and TCM-CRP deliver predictions that are 5–20% more accurate than those of JM. For patients 7 and 15, however, the prediction accuracy is 25% and 35% better, respectively. Compared to TCM and TCM-CRP, JM performs slightly better for patients 11, 14, 21 and 4, who favour JM by 5–12%. In 13 cases out of 23, the predictive performance of JM appears better than that of NM. The figure with the four week prediction horizon, with similar findings, is given in the Supplementary Fig. 1.

Table 2 (underlying data available in the Supplementary Dataset 3) shows the in-sample metrics. The in-sample RMSE and MAE are computed between the ‘fitted mean‘ and the observed leukocyte counts. For TCM and TCM-CRP, we refer to the fitted mean as the exponentiated filtered mean of the state variable L obtained by first estimating the model parameters from the patient’s full dataset and then running EKF with all leukocyte counts set to missing, conditional on the estimated parameter values. For JM, the fitted mean is simply the exponentiated solution of L conditional on the parameter vector estimated from the full patient dataset, and for NM, the fitted mean is the estimate of ${\mu }_{nm}$. The in-sample means of RMSE, MAE and the coverage probabilities are very similar for JM, TCM and TCM-CRP, with JM reaching a slightly better value for CP_50_. As expected, the point predictions of TCM, TCM-CRP and JM are better than those of NM.

**Table 2:** The in-sample metrics per model: means of coverage probability (CP), mean absolute error (MAE) and root mean squared error (RMSE).

	TCM	TCM-CRP	JM	NM
${\overline{{\rm{CP}}}}_{50}$	0.571 (0.08)	0.572 (0.08)	0.545 (0.07)	0.583 (0.11)
${\overline{{\rm{CP}}}}_{90}$	0.904 (0.03)	0.906 (0.03)	0.904 (0.03)	0.921 (0.03)
$\overline{{\rm{MAE}}}$	0.795 (0.31)	0.800 (0.31)	0.812 (0.32)	0.924 (0.36)
$\overline{{\rm{RMSE}}}$	1.195 (0.56)	1.203 (0.57)	1.199 (0.56)	1.304 (0.58)

Standard deviations are in parentheses.

For many patients, the in-sample fits of JM exhibit oscillating behaviour, which by visual inspection is not present in the datasets. An example is shown in Fig. 2, which plots the fit of JM with TCM. In contrast, the fit of TCM is smoother and only captures the average behaviour of the leukocyte counts. See Supplementary Figs. 2–24 for graphical comparisons for all of the patients. The fits of the models TCM, TCM-CRP and JM to the full patient datasets are also given in the Supplementary Dataset 4.

**Figure 2:**
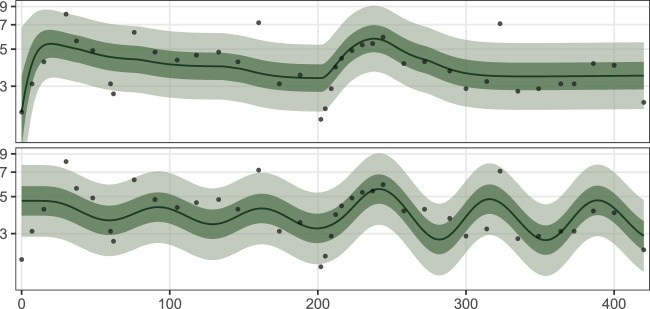
The models TCM (top) and JM (bottom) fit to the full dataset of patient 20 with time in days on the x-axis and leukocyte count on the y-axis. The fitted mean is the black line and probability intervals (50%, 90%) are plotted in green. The 6 MP dosage for the depicted patient was intensified incrementally to 50 mg during the first 200 days of treatment. After this, no dose was given for approximately 20 days. The dosage was then incrementally intensified back to 50 mg until treatment day 275 and kept constant until the end of the treatment. Further dose intensification was not possible due to low neutrophil counts.

**Figure 3:**
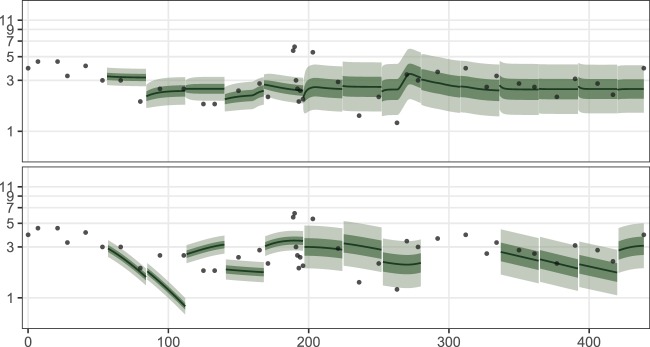
Predictions for patient 7 at each round of time series cross-validation with the four week prediction horizon for the models TCM (top) and JM (bottom). The plot for JM lacks predictions for treatment days 275–350, since the differential equation solver could not solve JM conditional on the parameter estimates found during optimisation. The 6 MP dosage for the depicted patient was intensified incrementally to 62.5 mg during the treatment. The dosage was not intensified further due to low neutrophil counts.

Inspecting the predictions made during TSC in a similar manner, we found that the weaker out-of-sample metrics for JM are partly explained by the fact that for many patients, the model produces unstable predictions especially in the beginning of the treatment when only a few measurements are available for parameter estimation. Figure 3 shows an example of this by plotting the predictions of JM and TCM from cross-validation with the four week prediction horizon. Here, the predictions of JM appear unstable until treatment day 175 while the predictions of TCM appear more consistent. The unstability is unfortunate, since in the beginning of the treatment there is a lot of uncertainty in how the treatment will affect the patient. Hence, good predictions in this period of treatment are particularly important. The figure also shows some of the estimation problems we faced with JM, since the differential equation solver was unable to make a prediction for treatment days 275–350. The similar figures for all the patients are shown in the Supplementary Figs. 25–47.

**Figure 4:**
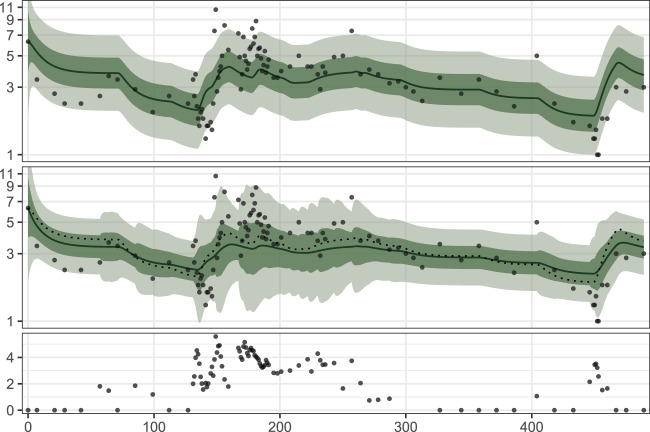
The fit of the models TCM (top) and TCM-CRP (middle) to the dataset of patient 4. The $\log (x+1)$-transformed CRP measurements are shown at the bottom. The dotted line in the plot for TCM-CRP is the fitted mean of TCM.

Based on Table 1 and Fig. 1, there appears to be little difference between the out-of-sample metrics of TCM and TCM-CRP, with TCM reaching slightly better values than TCM-CRP. However, TCM-CRP has an interesting property that is not visible in the predictive metrics. This is showcased in Fig. 4 where the fit of TCM is compared to that of TCM-CRP in the case of a patient with infections during the treatment. Here, accounting for the infection induced variability in the leukocyte count results in narrower probability intervals for TCM-CRP, when infection is not present. Furthermore, when compared to TCM, the fitted mean of TCM-CRP is slightly shifted away from leukocyte counts measured during infection, indicating that the model is downweighting observations that occur during infection.

## Discussion

In this work, we present two Bayesian nonlinear state space models, TCM and TCM-CRP, for predicting leukocyte counts during ALL MT. A predictive comparison between the models, and the model from the literature, JM, is then carried out. In prior works, predictive models for leukocyte counts during ALL MT have not been compared against each other according to their out-of-sample predictive performance. We argue that the development of predictive models should be guided by model comparison using out-of-sample metrics. Whenever possible, predictive models can also be validated by relating properties of the models to values available in the clinical literature. An approach like this was recently undertaken in a similar work^29^ related to acute myeloid leukaemia, where leukocyte count recovery times were used to discriminate between model candidates with similar predictive power.

The best-performing model according to our results, TCM, simplifies the model from the literature, JM, in the pharmacokinetic and the leukopoiesis model, and delivers a prediction accuracy competitive with JM. The simplification in the pharmacokinetic model results in a focus on the daily behaviour of the cytotoxicity induced by 6 MP, which is in contrast with the pharmacokinetic model of JM that models the pharmacokinetics in the hourly granularity. We believe that such a fine time scale is unnecessary, when predictions are required on a daily or weekly basis, as in the present application. Similarly, the simplification of the leukopoiesis model changes the focus from the daily granularity to the weekly, which is justified since the leukocyte counts are typically measured at this rate. Despite these simplifications, we argue that TCM still captures the most important features of the phenomenon: the effect of 6 MP on the level of cytotoxicity, and the effect of the cytotoxicity on the leukocyte counts. The simplifications also reduce the number of parameters to be estimated, which allows for robust estimation of the model with sparse clinical datasets.

In our experiments, we found that JM was difficult to estimate reliably with our heterogeneous dataset and we had issues with optimisation and prediction. In absence of better initial values for the parameters, we used the estimates reported by Jayachandran *et al*.^8,11^. If these estimates are far from adequate for the patients in our dataset, they can play a role in the estimation problems. However, in general almost any variation of the model we attempted to fit during the process of preparing this work had estimation problems for at least some patients. Perhaps related to the estimation problems, the computation time to produce the cross-validation results with the two week prediction horizon, for example, was roughly hundredfold for JM compared to that of TCM (16.55 hours vs. 0.15 hours).

A comment by a reviewer led us to realise that the initial values of the state variables of the JM leukopoiesis model seem to play a significant role on how the model performs. When we initialised them by estimating a common value for every state variable, there were less TSC rounds with convergence or prediction issues. However, this initialisation resulted in a lower predictive accuracy than the model presented, and hence we chose the steady state initialisation. Lately, the impact of the initialisation has also been noted in a similar work^29^, where models similar to the JM leukopoiesis model were investigated. It is possible that the alternative initialisation strategies found in the work might further improve the performance of JM.

Another noteworthy point regarding JM is that Jayachandran *et al*.^8,11^ had additional TGNRBC measurements in their dataset, which our dataset does not contain. Fitting the model without these measurements might have implications for the identifiability of the model, and hence the observed predictive performance. Furthermore, the dataset of Jayachandran *et al*.^11^ contains adults, and the pharmacokinetic profiles of adults and children differ. Allometric scaling^30^ could improve the model, and allow for more immediate interpretation.

The baseline model NM was found to perform on par with JM and have point predictive metrics not far from those of TCM. This is surprising, as the model does not account for the dosage administered to the patient. We suspect that the success of this over-simplified model might be explained by our data, where for many patients, the treatment was successful and the leukocyte counts were centered around a common value, which makes their mean a relatively good prediction. With data having little variation in the leukocyte counts and/or dosage, it is difficult to improve the predictive performance.

The model TCM-CRP extends TCM by incorporating C-reactive protein (CRP) measurements into the model as a surrogate for infections. To our knowledge, TCM-CRP is the first model to attempt the inclusion of infection information to a leukopoiesis model. In our dataset, 65% of the patients have at least one infection during their treatment (by counting patients who have at least one CRP measurement greater than or equal to 10 mg/L), highlighting the prevalence of infections in MT. Furthermore, as there is an evident relationship between CRP and the leukocyte count (see Fig. 4), we argue that infections should be accounted for in ALL MT predictive modelling. In previous works, patients with infections during the treatment have been excluded from analysis^8,9^.

Figure 4 shows a promising fit of TCM-CRP, but in general the parameter estimates computed for the model during TSC were similar to those of TCM, leading to similar predictions. This is likely because the state variable *V* in TCM-CRP does not directly influence the mean of the state variable *L*, but controls its variability instead. We modelled infection this way, because the relationship between CRP and the leukocyte count appears hard to predict: at least in our data, elevated CRP seems to be associated with both increased and decreased leukocyte counts, with no apparent pattern. This is not surprising, since it is well known that CRP is nonspecific and can exhibit variable behaviour in different kinds of inflammatory states. Hence, our modelling strategy for infections did not aim to utilise CRP as a regressor (or predictor) for leukocyte counts, but rather to improve the robustness of the model against infections by downweighting the outlying leukocyte counts when CRP is elevated. Our hope was that this would result in a model that better predicts data measured when no infection is present. Based on the obtained results, this was not entirely successful, perhaps due to the proposed Ornstein-Uhlenbeck model and possibly the functional form of *σ*_*L*_ being inadequately specified. The approximate nature of EKF can also play a role here, and better results for TCM-CRP could possibly be obtained by using more accurate estimation methods, such as particle Markov chain Monte Carlo^31^.

The existing models predicting leukocyte counts during ALL MT use ordinary differential equation models^8,9^. In contrast to that approach, the nonlinear state space models we use allow for additional stochasticity in the state equation of the model, which we believe helps account for unmodelled variations in the data more accurately.

The leukopoiesis models of Jayachandran *et al*.^8^ and Le *et al*.^9^ extend the well-known 5-compartment structure introduced by Friberg *et al*.^12^, for 6 MP (and MTX). It is worth mentioning that the chemotherapy drugs considered by Friberg *et al*. do not include 6 MP (or MTX), and are given in pulses, which is in contrast with the continuous low-dose administration of 6 MP in ALL MT. This may explain why our simpler one-compartment leukopoiesis model provided an improved predictive model in our experiments, and suggests that the commonly used 5-compartment model might not be optimal for all applications.

Little is known about the adequacy of pharmacokinetic models of 6 MP too, as datasets with recorded 6 MP doses and metabolites are rare and sparse, making model validation difficult. We are only aware of the works of Jayachandran *et al*.^8,11^ and Hawwa *et al*.^32^ where the dataset contained data on both administered 6 MP doses and TGNRBC. Furthermore, out-of-sample model comparison was only performed by Hawwa *et al*. Although TGNRBC was previously found to be associated with myelosuppression^33^, later research has shown TGNRBC to be only weakly related to levels of DNA-thioguanine (TGNDNA), the main mediator of the cytotoxicity of 6 MP^34^. Hence, modelling TGNRBC as the end point of the pharmacokinetic model might not provide optimal predictions when the model is used in conjunction with a leukopoiesis model.

Recently, there has been increased interest in TGNDNA, as a study has found higher TGNDNA concentrations associated with improved relapse-free survival^2^ and dosage could potentially be guided better by monitoring TGNDNA concentrations, as factors such as age, ethnicity and time of year confound the leukocyte counts^35,36^. However, to our knowledge, pharmacokinetic models for 6 MP with TGNDNA as the end point have not yet emerged and present an interesting prospect for future research regarding predictive modelling in the context of ALL MT. Moreover, if data with TGNDNA concentrations and leukocyte counts were available, the modelling framework of nonlinear state space models used in this work could readily incorporate the metabolite measurements into the model, and would in theory allow for the simultaneous prediction of the leukocyte count and the TGNDNA concentration, providing the clinician with extra information for decision-making.

In this work, we considered modelling the leukocyte counts based on 6 MP dosage only. We did not attempt to include MTX into our models, because the 6 MP and MTX dosages are strongly linked in our data, making reliable estimation of a joint model difficult. The concurrent work of Le *et al*.^9^ incorporated patient MTX doses into their leukopoiesis model. While a comparison of the model to a model without MTX was not shown, incorporating MTX is likely an important step forward in ALL MT predictive modelling. However, we note that the rationale of MTX dosage in ALL MT is mainly that the drug increases the bioavailability of 6 MP^37,38^, and only partially the cytotoxic effects of the drug itself. Hence, rather than incorporating the metabolites of MTX into the function ${e}_{drug}$ as was done by Le *et al*., our intuition is that the MTX metabolites should rather be a covariate in the pharmacokinetic model for 6 MP, perhaps related to the value of the parameter ${e}_{tgn}$ in (7) or similar in another model.

Another interesting work in the literature is the work of Hawwa *et al*.^32^, who investigated population pharmacokinetic models for 6 MP. The authors incorporated patient thiopurine methyltransferase (TPMT) genotype and BSA as covariates into their model and found that both variables reduced the interindividual variability in the model parameters significantly. We did not include the TPMT genotype to our pharmacokinetic model as the data were missing for 13 of the 23 patients, and of the remaining patients, 9 were of TPMT wildtype, only one was TPMT heterozygous and there were no TPMT homozygotes. Hence, with the current data available, our model is representative of patients who are of TPMT wildtype, the genotype that covers 86–97%^39^ of the population.

Combining our work with the work of Le *et al*.^9^ and Hawwa *et al*.^32^, it is possible to envision a model with the important covariates taken into account, improving the leukocyte count predictions. However, availability and sparseness of datasets remains a problem. Further improvements to the predictive performance could likely be obtained with hierarchical models linking the parameter vectors of the individual patients with hyperparameters. Such joint modelling has, to our knowledge, only been conducted in the context of ALL MT by Hawwa *et al*. with their pharmacokinetic model. In the course of preparing this work, we attempted to fit such models, but faced unresolvable computational problems likely due to the lack of 6 MP metabolite measurements in the dataset. Simpler joint models assuming the same values for a subset of parameters across patients were estimatable, but did not produce better predictive results than fitting the models to each dataset individually, likely due to the high interindividual variability in the parameters.

## Supplementary information

Supplementary information

Dataset 1

Dataset 2

Dataset 3

Dataset 4

## Data Availability

All data generated or analysed during this study are included in this published article (and its Supplementary Information files).
