# Supplementary material for: Prediction of leukocyte counts during paediatric acute lymphoblastic leukaemia maintenance therapy
Source: Sci Rep. 2019 Dec 2;9:18076. doi: 10.1038/s41598-019-54492-5 (PMC6889389; doi:10.1038/s41598-019-54492-5)
Supplement: Supplementary file 1 — Supplementary information [file 41598_2019_54492_MOESM1_ESM.pdf]

# PREDICTION OF LEUKOCYTE COUNTS DURING PAEDIATRIC ACUTE LYMPHOBLASTIC LEUKAEMIA MAINTENANCE THERAPY, SUPPLEMENTARY INFORMATION

SANTERI KARPPINEN, OLLI LOHI, AND MATTI VIHOLA

## 1. Supplementary Tables

Supplementary Table 1. Glossary for the state variables appearing in the models JM, TCM and TCM-CRP.

| State variable | Description | Unit |
|---|---|---|
| $X_{gut}$ | 6MP in the gut | pmol |
| $X_{plasma}$ | 6MP in the plasma | pmol |
| $X_{tgn}$ | $\text{TGN}_{\text{RBC}}$ concentration | pmol / $8\times10^8$ RBC |
| $M$ | Cytotoxicity induced by 6MP | per day |
| $\exp(S)$ | Progenitor cells in the bone marrow | $10^9$/L |
| $\exp(C^{(i)})$ | Maturing cells in compartment $i \in \{1,2,3\}$ | $10^9$/L |
| $\exp(L)$ | Leukocytes in circulation | $10^9$/L |
| $\exp(V) - 1$ | Level of infection | mg/L |

Supplementary Table 2. Glossary for the parameters appearing in JM. The fixed parameter values are from [1, 2]. Parameter $v_{cm}$ is fixed to 1.0 as in [1] for no value was given in [2].

| Parameter | Description | Unit | Value |
|---|---|---|---|
| $k_{cm}$ | 6MP to $\text{TGN}_{\text{RBC}}$ conversion rate | pmol 6MP per day | estimated |
| $k_{tr}$ | Leukopoiesis transfer rate | per day | estimated |
| $k_L$ | Leukocyte elimination rate | per day | estimated |
| $k_{pl}^{max}$ | Maximum proliferation rate | per day | estimated |
| $e_{max}$ | $\text{TGN}_{\text{RBC}}$ max. effect on progenitor cells | per day | estimated |
| $\gamma$ | Feedback shape parameter | - | estimated |
| $\sigma_{leuk}$ | Log-leukocyte measurement std. | - | estimated |
| $k_{ab}$ | 6MP absorption rate | per day | 4.2 |
| $k_{el}$ | 6MP elimination rate | per day | 3.8 |
| $k$ | Michaelis-Menten constant | pmol | 15.11 |
| $k_{me}$ | $\text{TGN}_{\text{RBC}}$ elimination rate | per day | 0.08 |
| $\rho$ | Feedback parameter | $10^9$/L | 8.2 |
| $e_{C50}$ | $\text{TGN}_{\text{RBC}}$ half effect on progenitor cells | pmol / $8\times10^8$ RBC | 84.0 |
| $v_{cm}$ | Stoichiometric coefficient for $\text{TGN}_{\text{RBC}}$ | pmol per pmol 6MP / $8\times10^8$ RBC | 1.0 |

Supplementary Table 3. Glossary for the parameters appearing in TCM. The fixed parameter values are from [1, 2], with the exception of $\sigma_{leuk}$, the value of which is a literature value for the accuracy of measuring neutrophil counts [3].

| Parameter | Description | Unit | Value |
|---|---|---|---|
| $h$ | BSA-scaled 6MP half effect on $M$ | mg / $m^2$ | estimated |
| $k_{pl}^{max}$ | Maximum proliferation rate | per day | estimated |
| $k_L$ | Leukocyte elimination rate | per day | estimated |
| $e_{tgn}$ | BSA-scaled 6MP maximum effect on $M$ | per day | estimated |
| $\sigma_L$ | Leukopoiesis std. | - | estimated |
| $k_{me}$ | $M$ elimination rate | per day | 0.08 |
| $\rho$ | Feedback parameter | $10^9$/L | 8.2 |
| $\sigma_{leuk}$ | Log-leukocyte measurement std. | - | 0.057 |
| $\gamma$ | Feedback shape parameter | - | 0.4368 |

Supplementary Table 4. Glossary for the parameters appearing in TCM-CRP. The parameters not mentioned are as in TCM.

| Parameter | Description | Value |
|---|---|---|
| $\sigma_L^0$ | Nominal leukocyte std. | estimated |
| $\sigma_{ou}$ | Ornstein-Uhlenbeck std. | 0.4268 |
| $\theta_{ou}$ | Ornstein-Uhlenbeck rate of mean reversion | 0.0805 |
| $\beta_{crp}$ | Variance inflation coefficient | 0.1915 |
| $\sigma_{crp}$ | $\log(\mathrm{CRP}+1)$-measurement std. | 0.1 |

Supplementary Table 5. Glossary for the parameters appearing in NM.

| Parameter | Description | Value |
|---|---|---|
| $\mu_{nm}$ | Mean leukocyte count | estimated |
| $\sigma_{nm}$ | Leukocyte standard deviation | estimated |

## 2. Supplementary figures

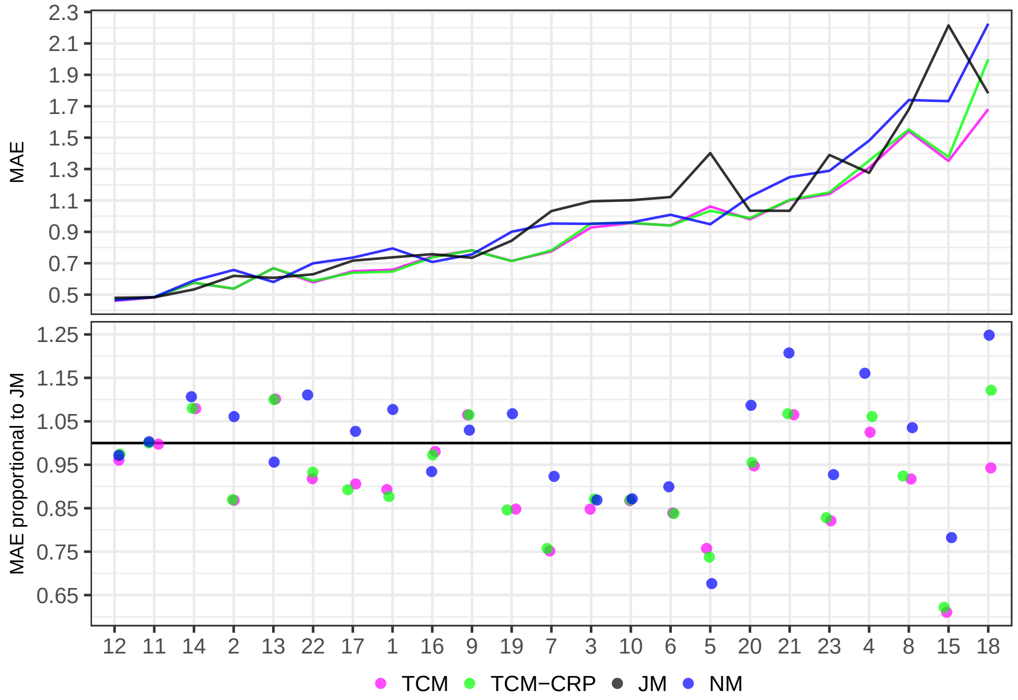


Supplementary Figure 1. The patientwise out-of-sample mean absolute error (top) and the out-of-sample mean absolute errors of the models TCM, TCM-CRP and NM proportional to the mean absolute errors of JM (bottom). The black line in the bottom plot depicts the line of equal predictive accuracy with JM. The out-of-sample values are from time series cross-validation with the four week prediction horizon. Each model is represented by a color. The patients have been ordered with increasing mean MAE over the models.

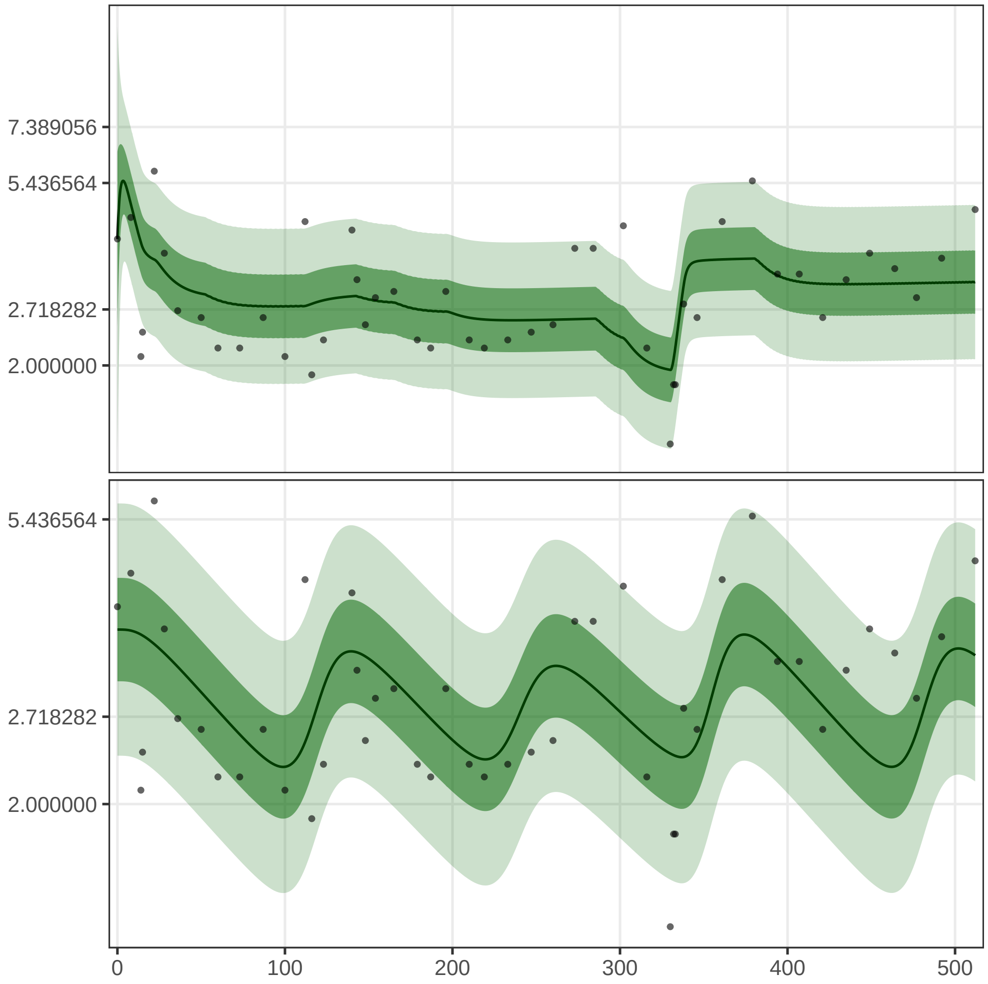


Supplementary Figure 2. The models TCM (top) and JM (bottom) fit to the full dataset of patient 1 with time in days on the x-axis and leukocyte count on the y-axis. The fitted mean is the black line and probability intervals (50%, 90%) are plotted in green.

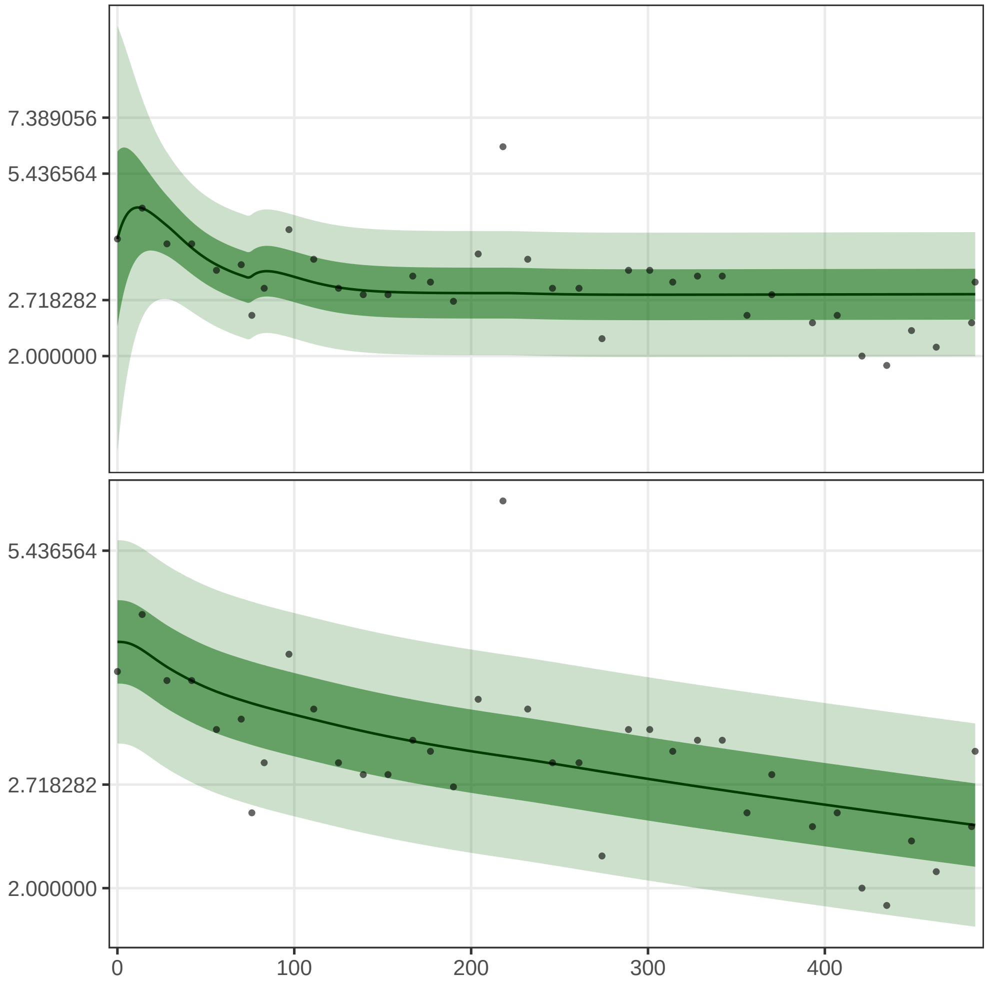


Supplementary Figure 3. The models TCM (top) and JM (bottom) fit to the full dataset of patient 2 with time in days on the x-axis and leukocyte count on the y-axis. The fitted mean is the black line and probability intervals (50%, 90%) are plotted in green.

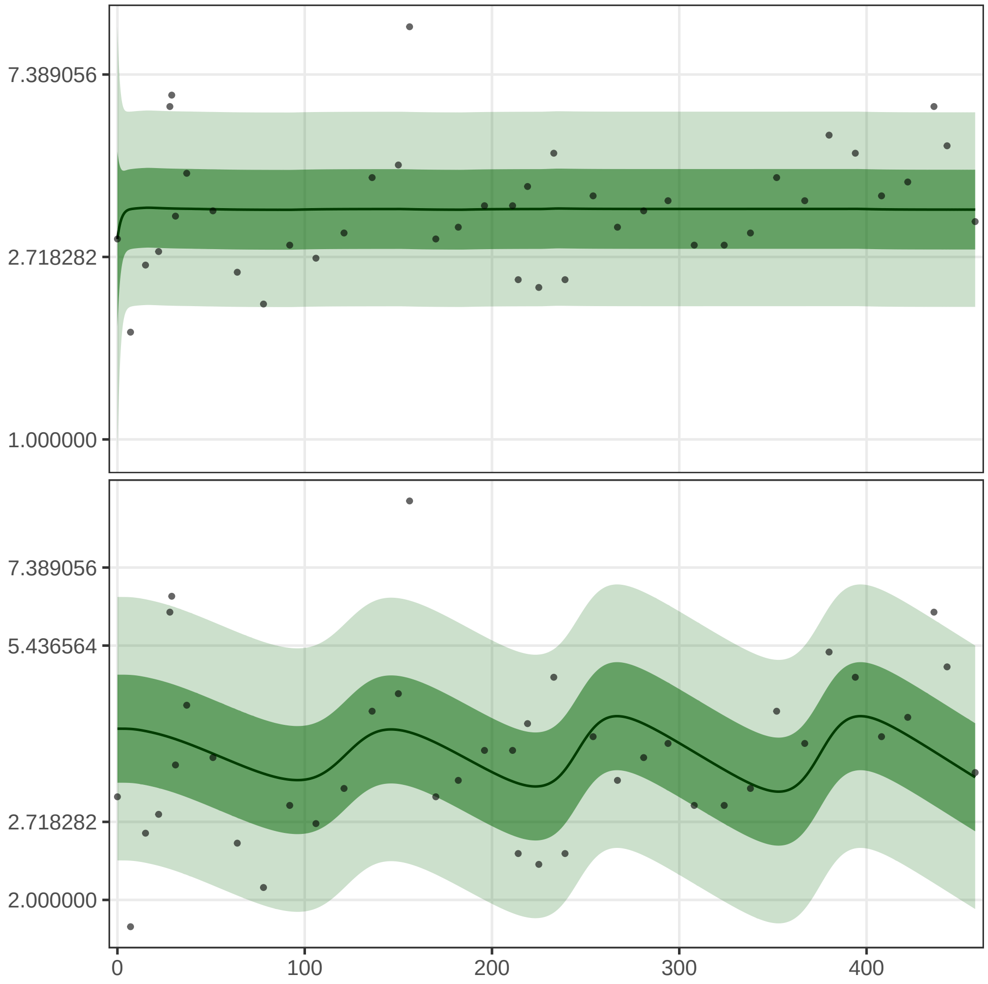


Supplementary Figure 4. The models TCM (top) and JM (bottom) fit to the full dataset of patient 3 with time in days on the x-axis and leukocyte count on the y-axis. The fitted mean is the black line and probability intervals (50%, 90%) are plotted in green.

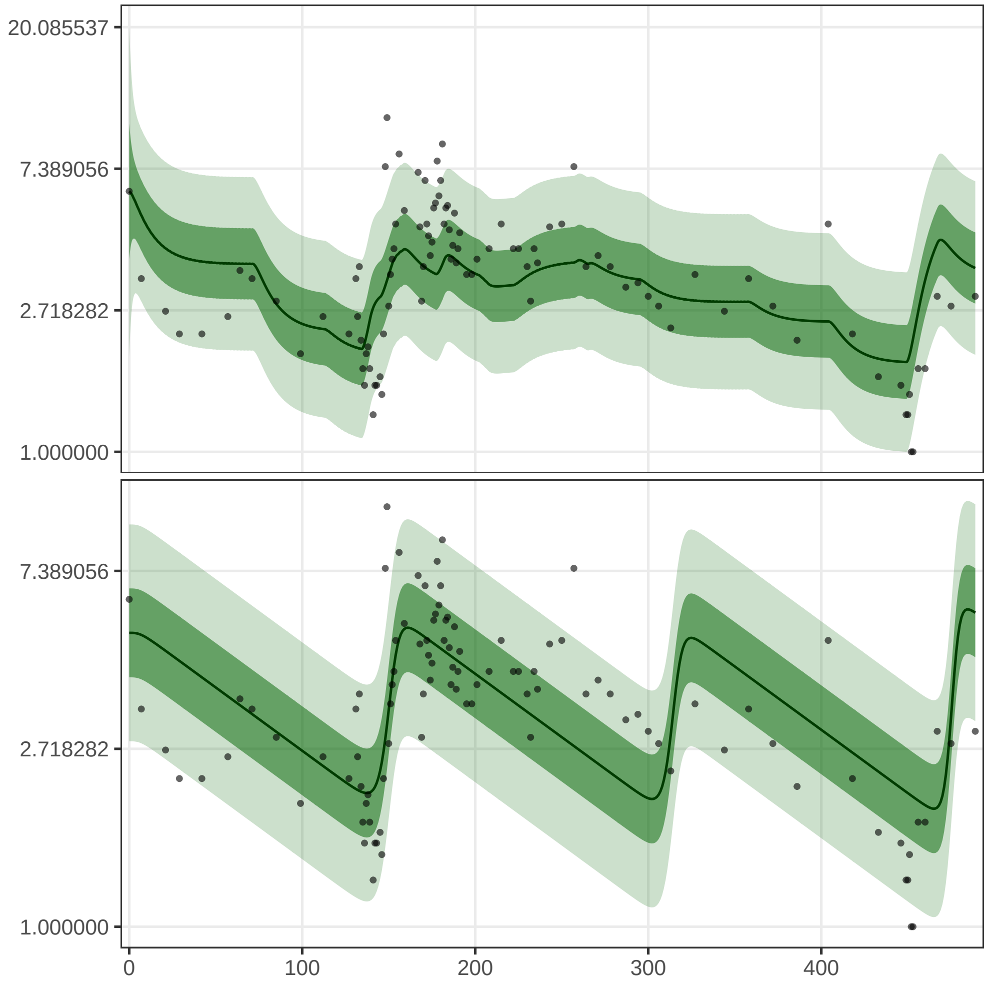


Supplementary Figure 5. The models TCM (top) and JM (bottom) fit to the full dataset of patient 4 with time in days on the x-axis and leukocyte count on the y-axis. The fitted mean is the black line and probability intervals (50%, 90%) are plotted in green.

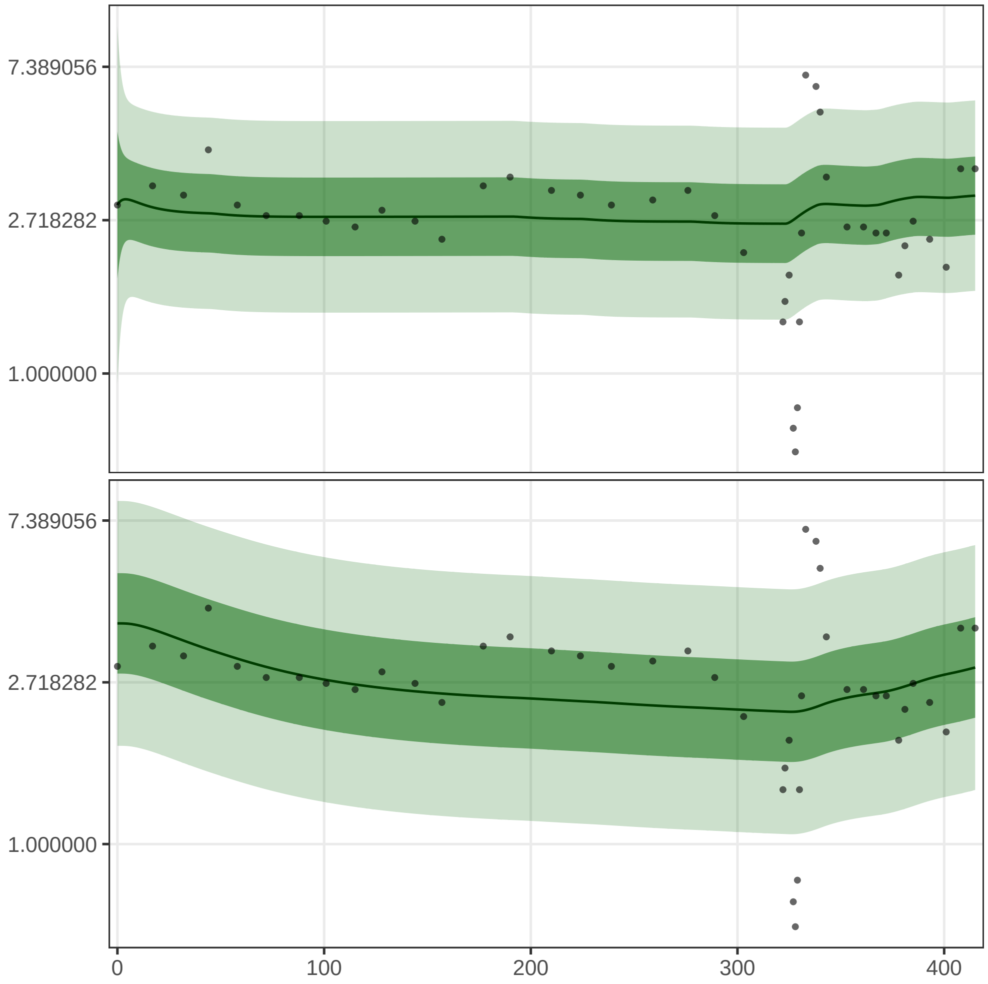


Supplementary Figure 6. The models TCM (top) and JM (bottom) fit to the full dataset of patient 5 with time in days on the x-axis and leukocyte count on the y-axis. The fitted mean is the black line and probability intervals (50%, 90%) are plotted in green.

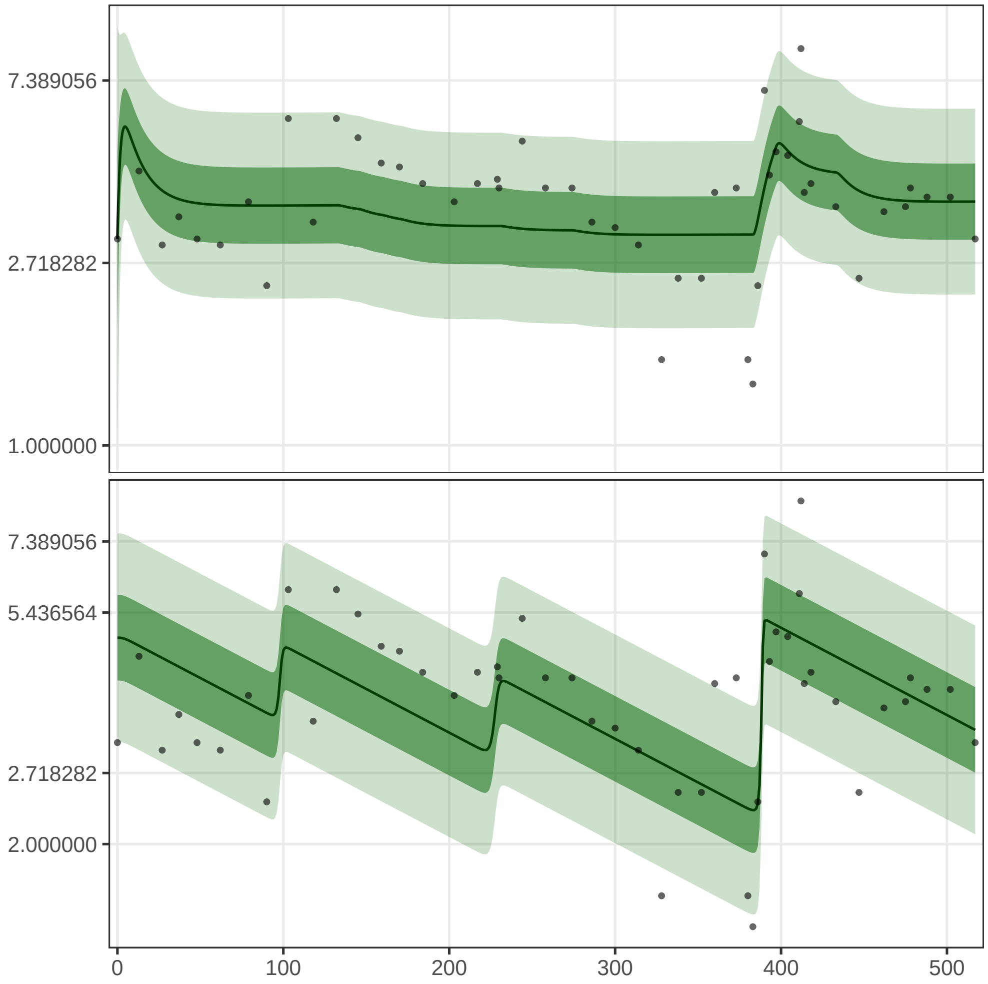


Supplementary Figure 7. The models TCM (top) and JM (bottom) fit to the full dataset of patient 6 with time in days on the x-axis and leukocyte count on the y-axis. The fitted mean is the black line and probability intervals (50%, 90%) are plotted in green.

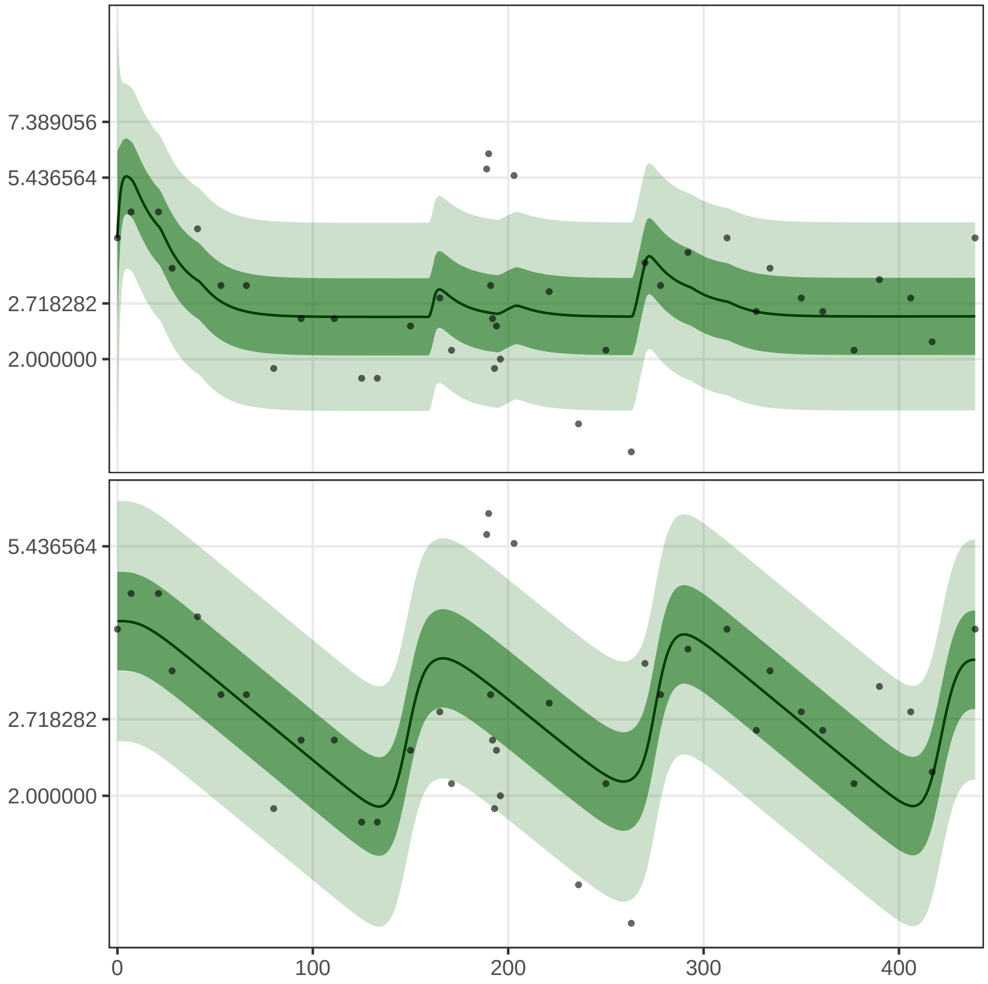


SUPPLEMENTARY FIGURE 8. The models TCM (top) and JM (bottom) fit to the full dataset of patient 7 with time in days on the x-axis and leukocyte count on the y-axis. The fitted mean is the black line and probability intervals (50%, 90%) are plotted in green.

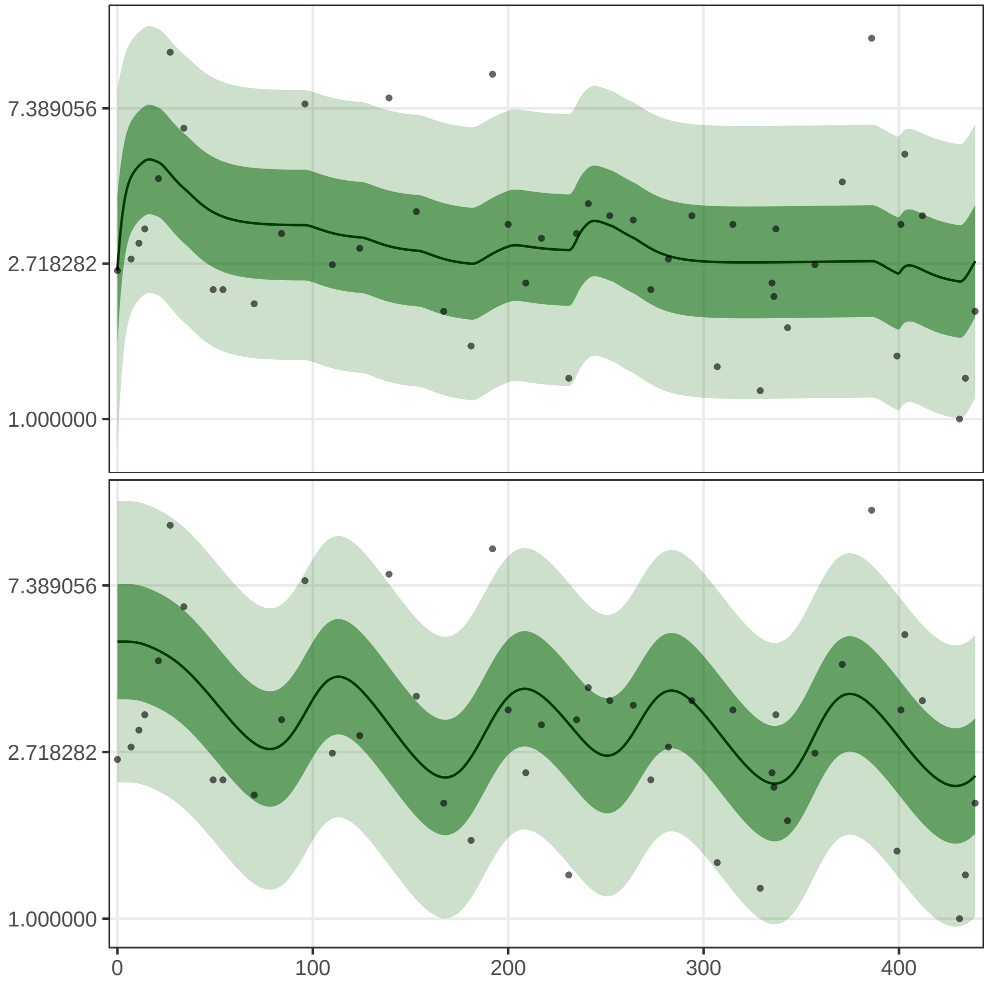


Supplementary Figure 9. The models TCM (top) and JM (bottom) fit to the full dataset of patient 8 with time in days on the x-axis and leukocyte count on the y-axis. The fitted mean is the black line and probability intervals (50%, 90%) are plotted in green.

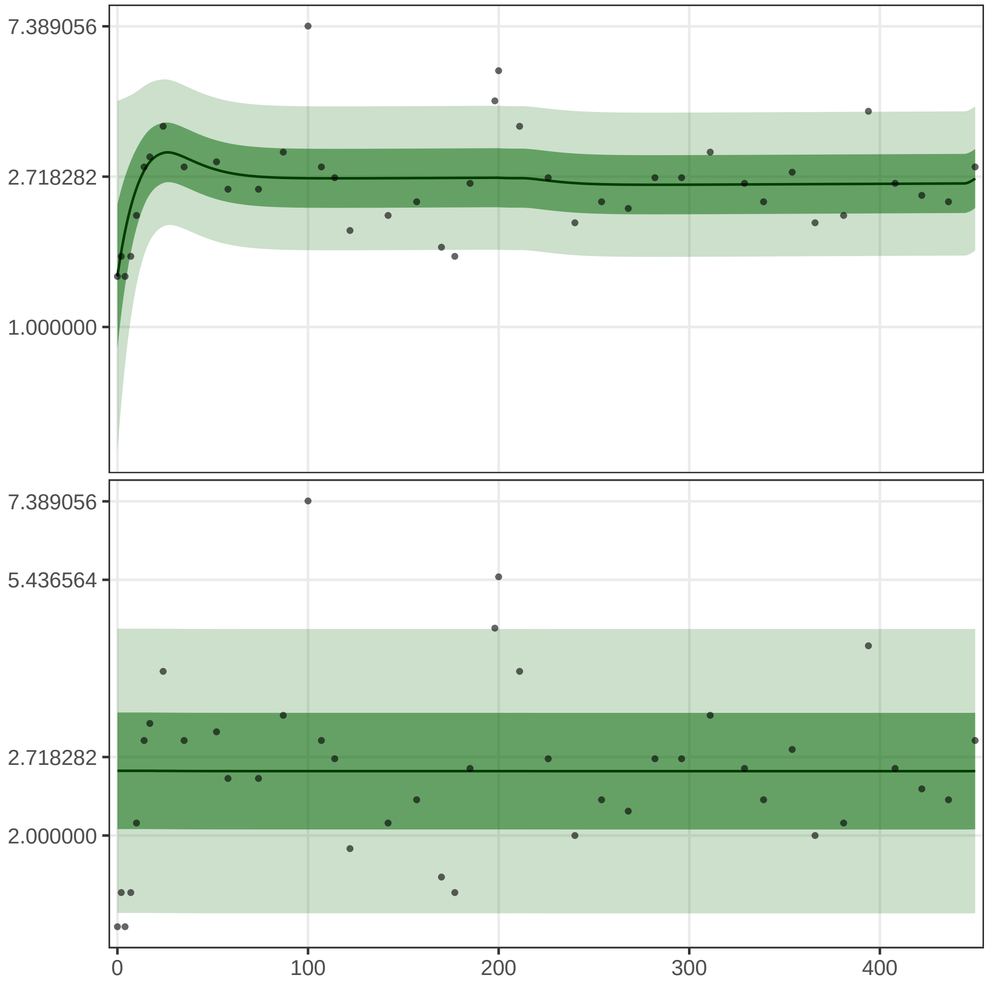


Supplementary Figure 10. The models TCM (top) and JM (bottom) fit to the full dataset of patient 9 with time in days on the x-axis and leukocyte count on the y-axis. The fitted mean is the black line and probability intervals (50%, 90%) are plotted in green.

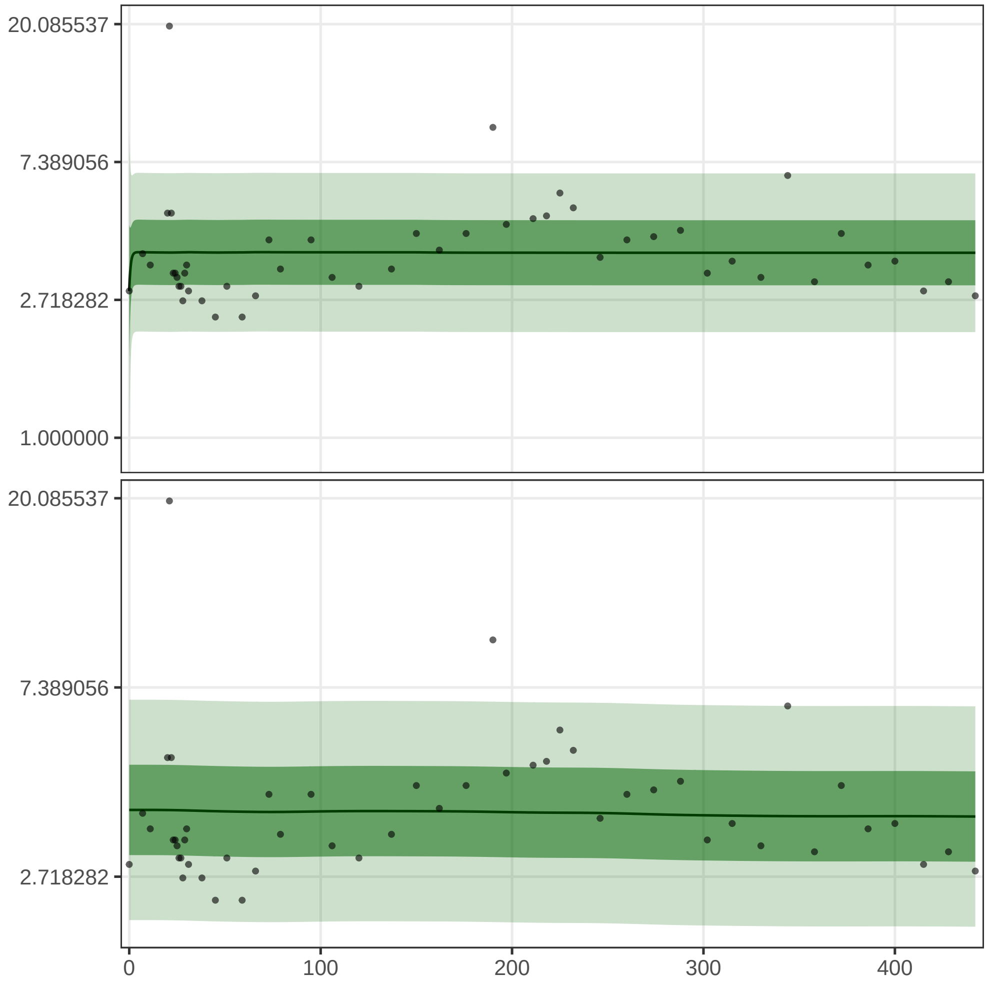


Supplementary Figure 11. The models TCM (top) and JM (bottom) fit to the full dataset of patient 10 with time in days on the x-axis and leukocyte count on the y-axis. The fitted mean is the black line and probability intervals (50%, 90%) are plotted in green.

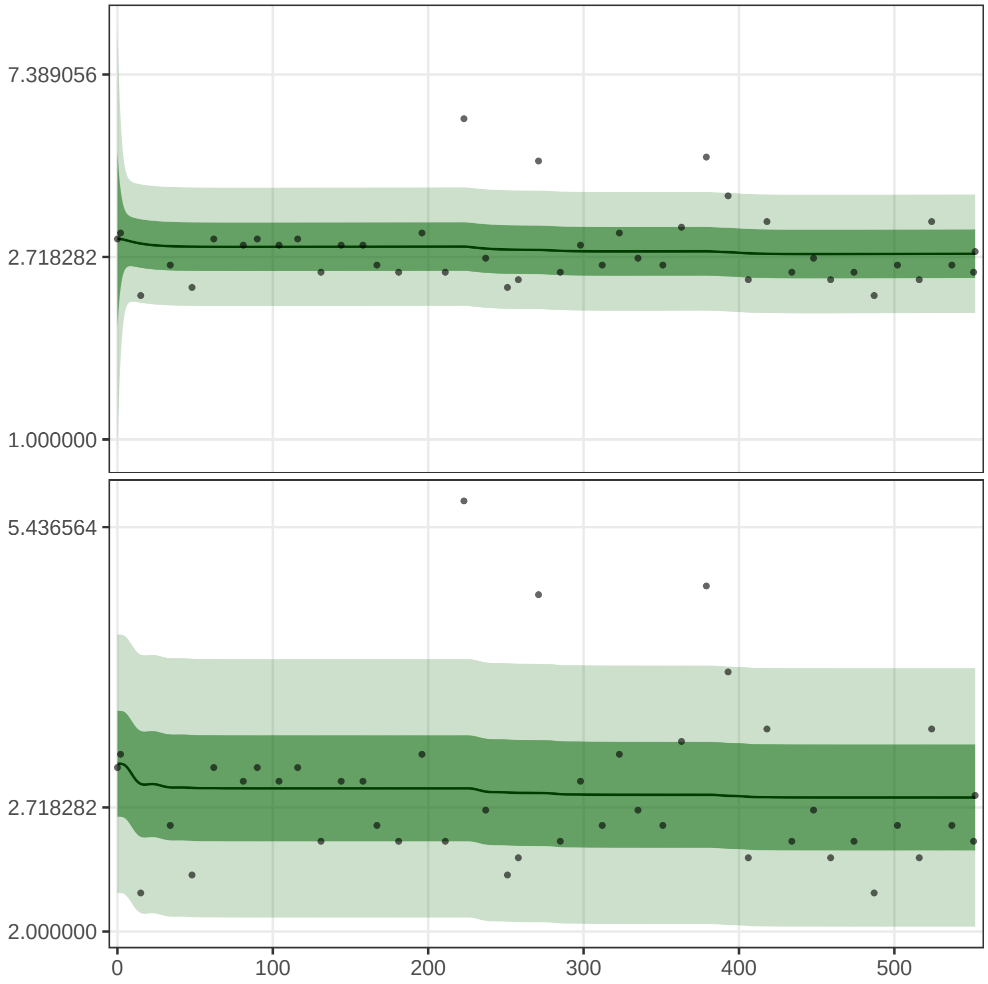


Supplementary Figure 12. The models TCM (top) and JM (bottom) fit to the full dataset of patient 11 with time in days on the x-axis and leukocyte count on the y-axis. The fitted mean is the black line and probability intervals (50%, 90%) are plotted in green.

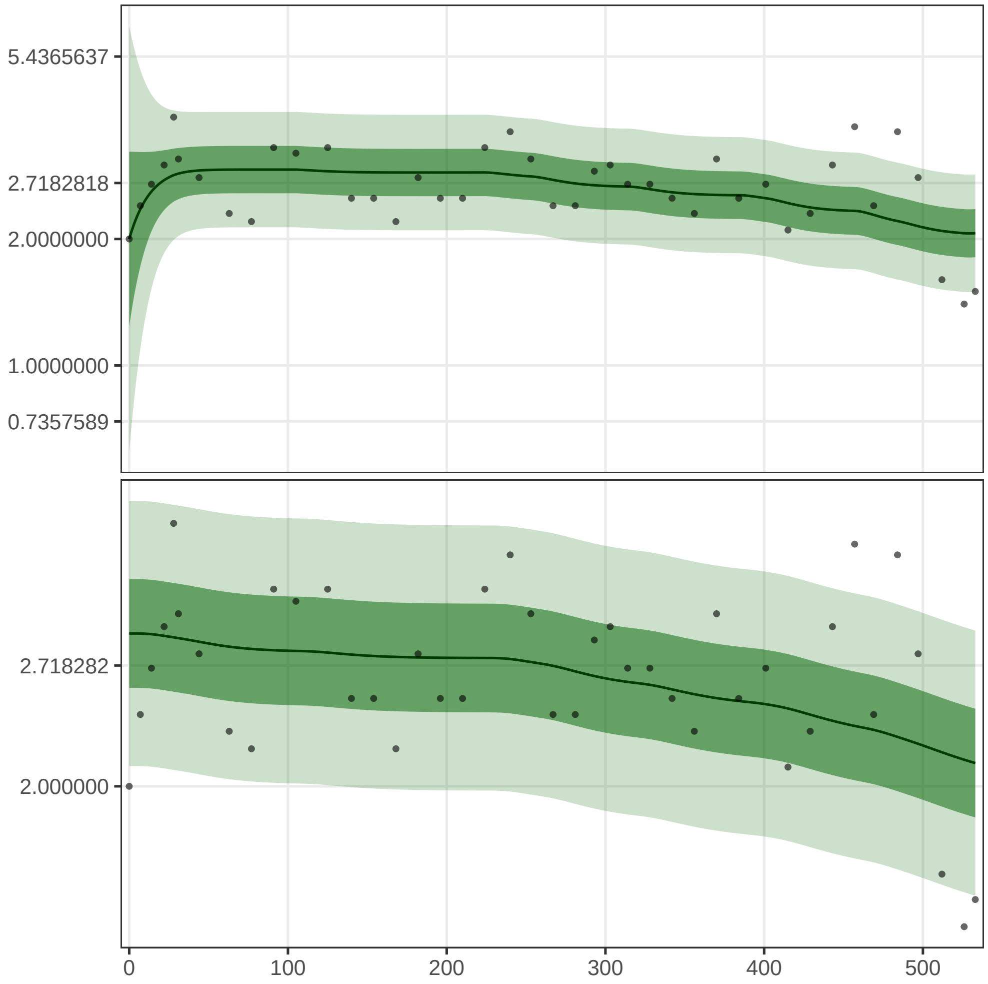


Supplementary Figure 13. The models TCM (top) and JM (bottom) fit to the full dataset of patient 12 with time in days on the x-axis and leukocyte count on the y-axis. The fitted mean is the black line and probability intervals (50%, 90%) are plotted in green.

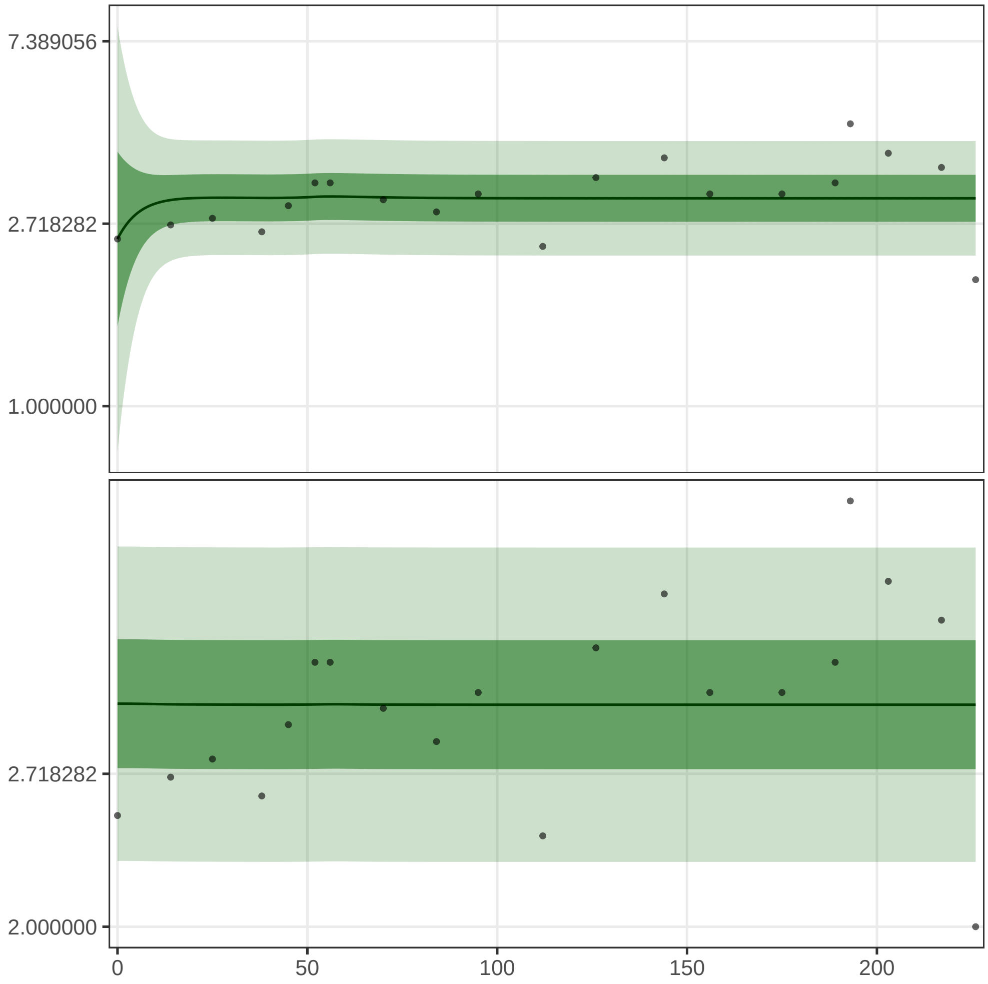


Supplementary Figure 14. The models TCM (top) and JM (bottom) fit to the full dataset of patient 13 with time in days on the x-axis and leukocyte count on the y-axis. The fitted mean is the black line and probability intervals (50%, 90%) are plotted in green.

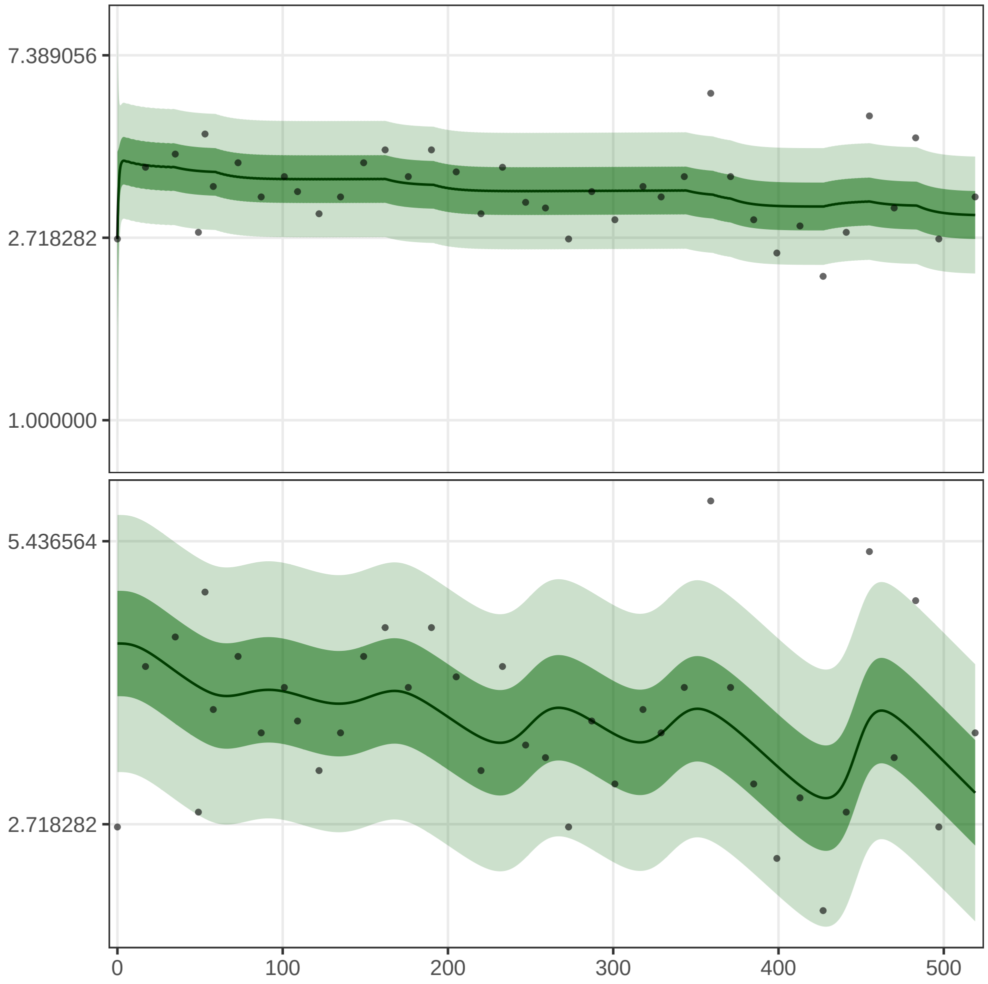


Supplementary Figure 15. The models TCM (top) and JM (bottom) fit to the full dataset of patient 14 with time in days on the x-axis and leukocyte count on the y-axis. The fitted mean is the black line and probability intervals (50%, 90%) are plotted in green.

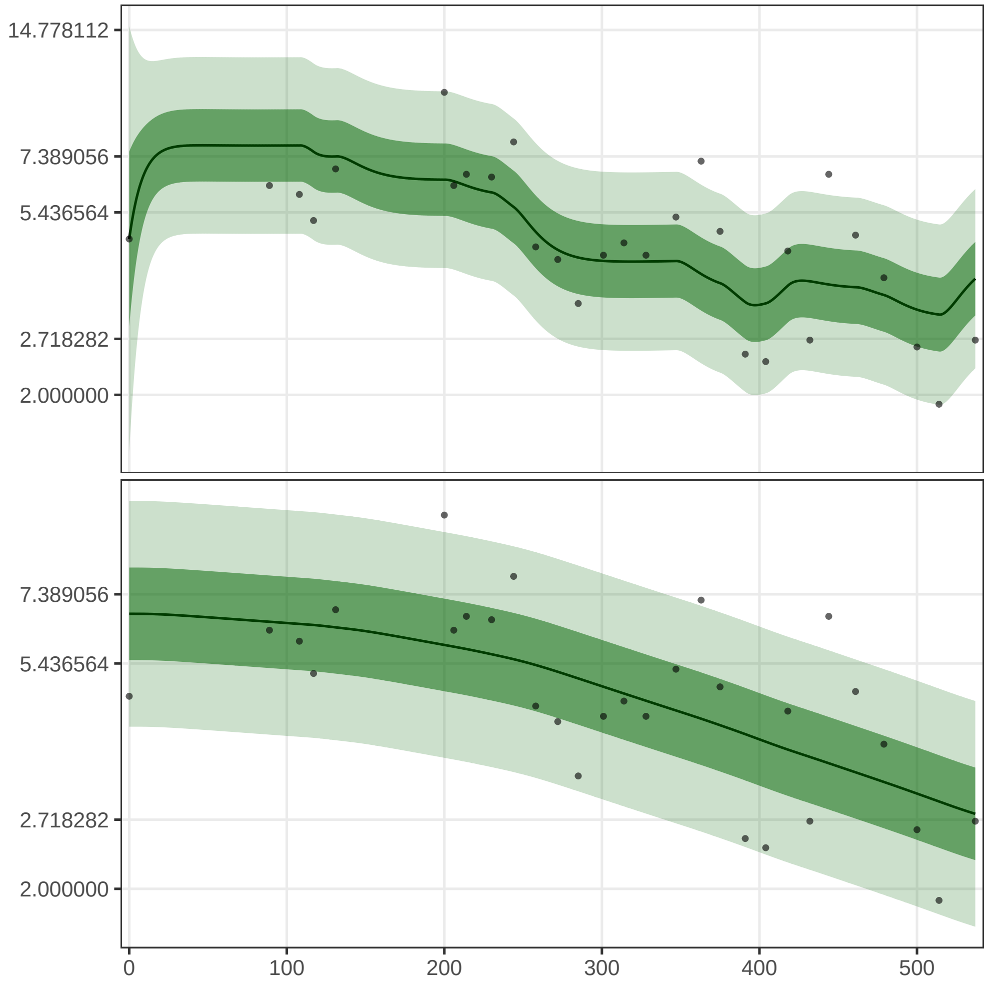


Supplementary Figure 16. The models TCM (top) and JM (bottom) fit to the full dataset of patient 15 with time in days on the x-axis and leukocyte count on the y-axis. The fitted mean is the black line and probability intervals (50%, 90%) are plotted in green.

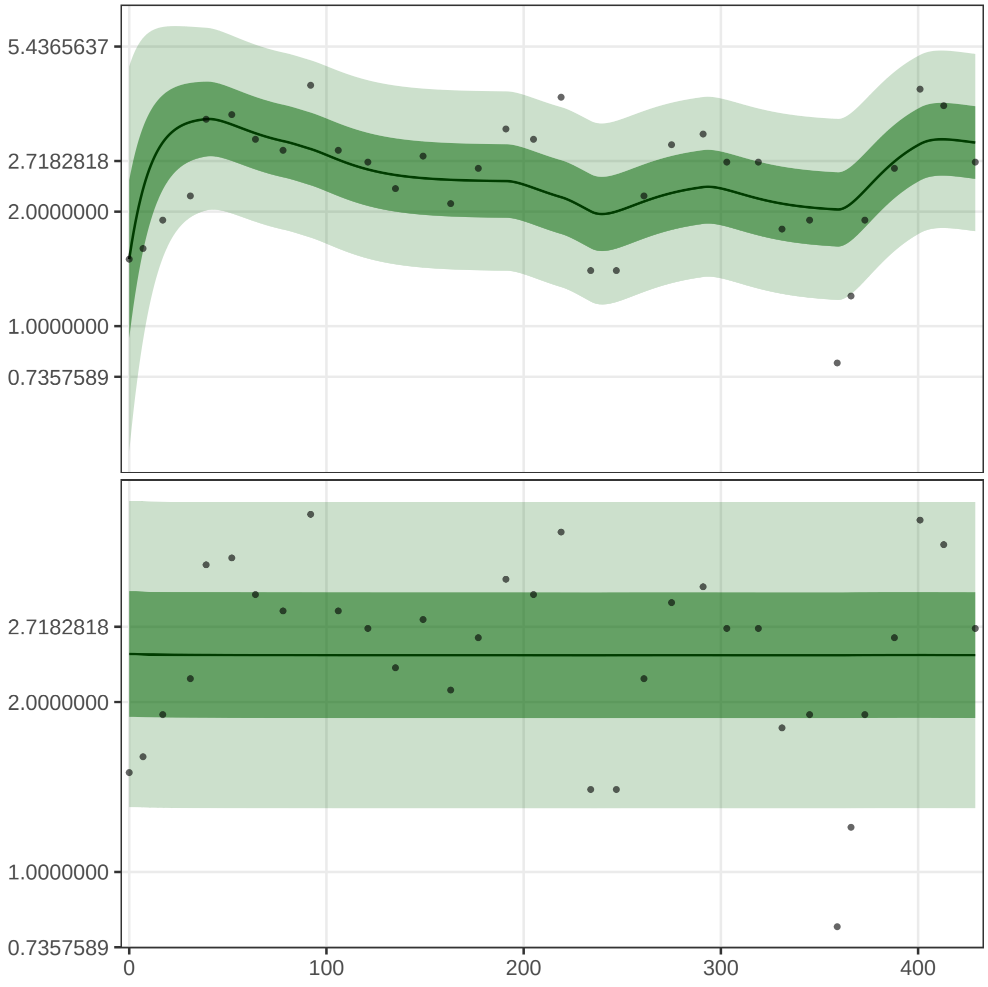


Supplementary Figure 17. The models TCM (top) and JM (bottom) fit to the full dataset of patient 16 with time in days on the x-axis and leukocyte count on the y-axis. The fitted mean is the black line and probability intervals (50%, 90%) are plotted in green.

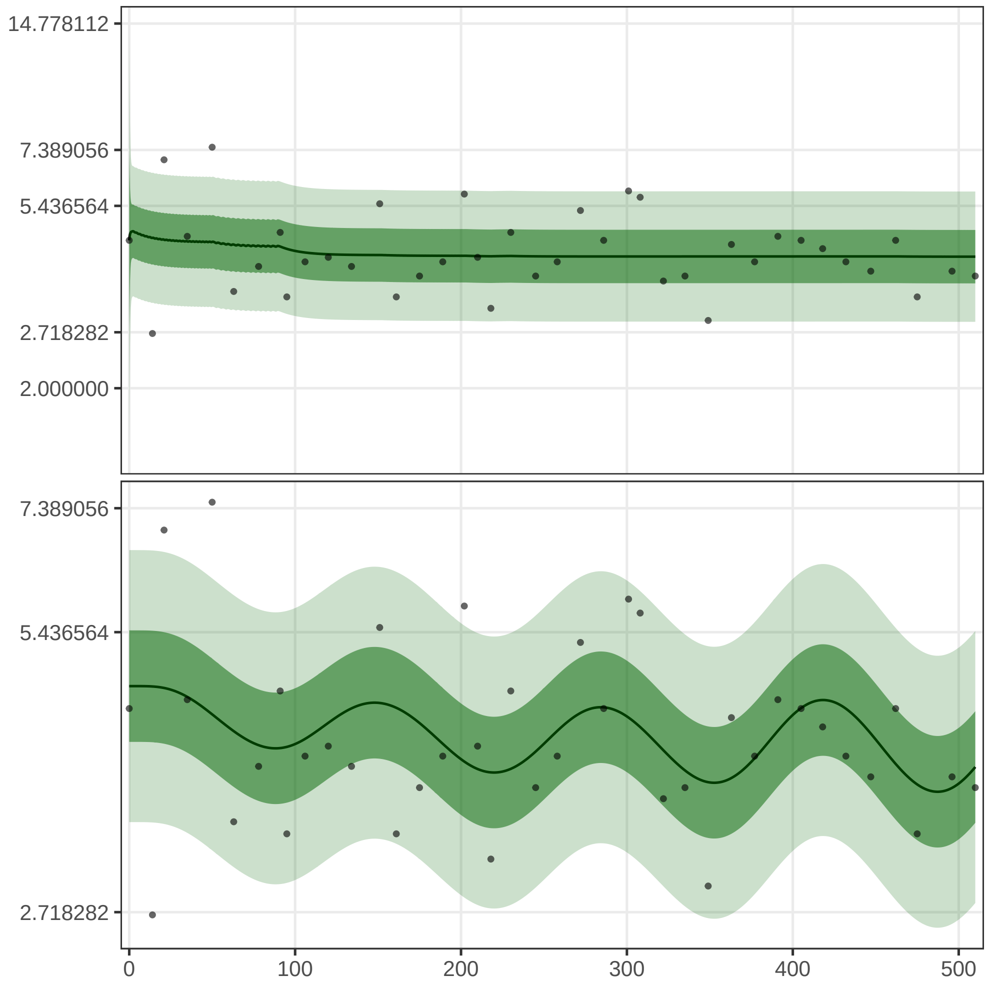


Supplementary Figure 18. The models TCM (top) and JM (bottom) fit to the full dataset of patient 17 with time in days on the x-axis and leukocyte count on the y-axis. The fitted mean is the black line and probability intervals (50%, 90%) are plotted in green.

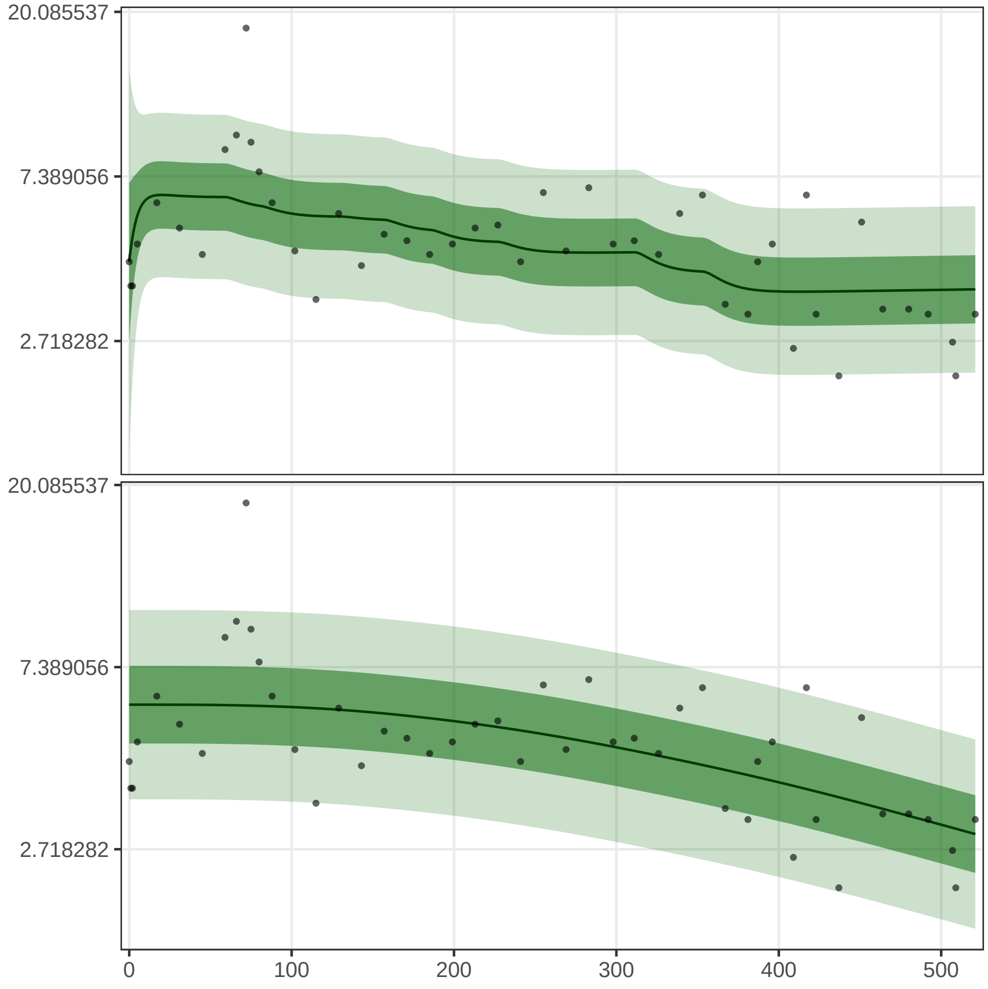


SUPPLEMENTARY FIGURE 19. The models TCM (top) and JM (bottom) fit to the full dataset of patient 18 with time in days on the x-axis and leukocyte count on the y-axis. The fitted mean is the black line and probability intervals (50%, 90%) are plotted in green.

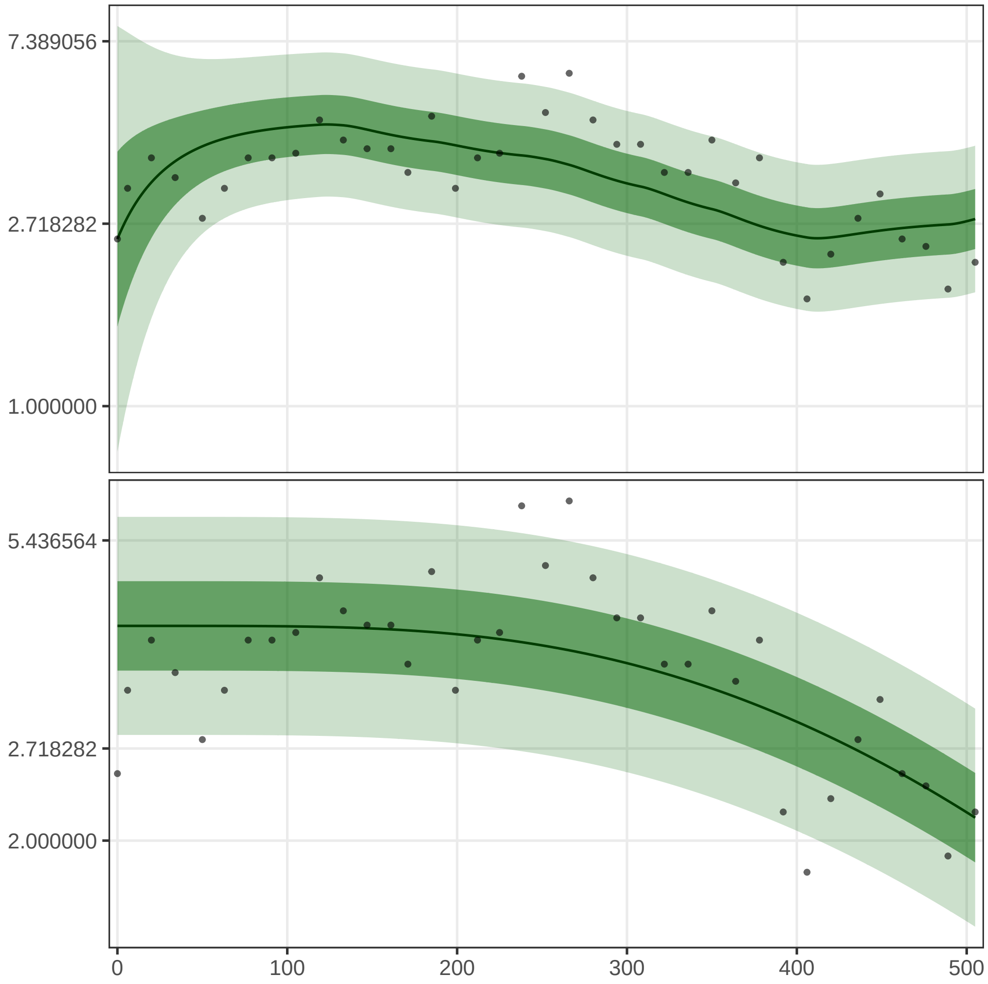


Supplementary Figure 20. The models TCM (top) and JM (bottom) fit to the full dataset of patient 19 with time in days on the x-axis and leukocyte count on the y-axis. The fitted mean is the black line and probability intervals (50%, 90%) are plotted in green.

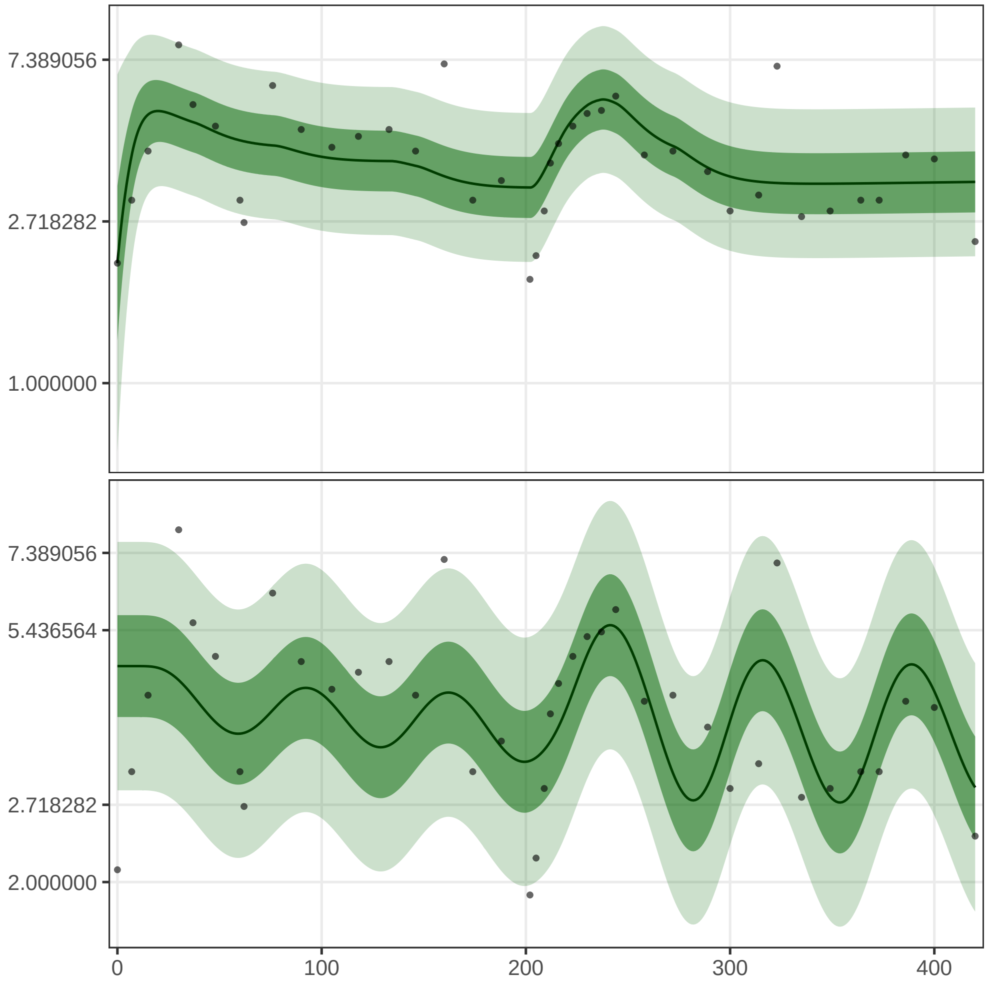


Supplementary Figure 21. The models TCM (top) and JM (bottom) fit to the full dataset of patient 20 with time in days on the x-axis and leukocyte count on the y-axis. The fitted mean is the black line and probability intervals (50%, 90%) are plotted in green.

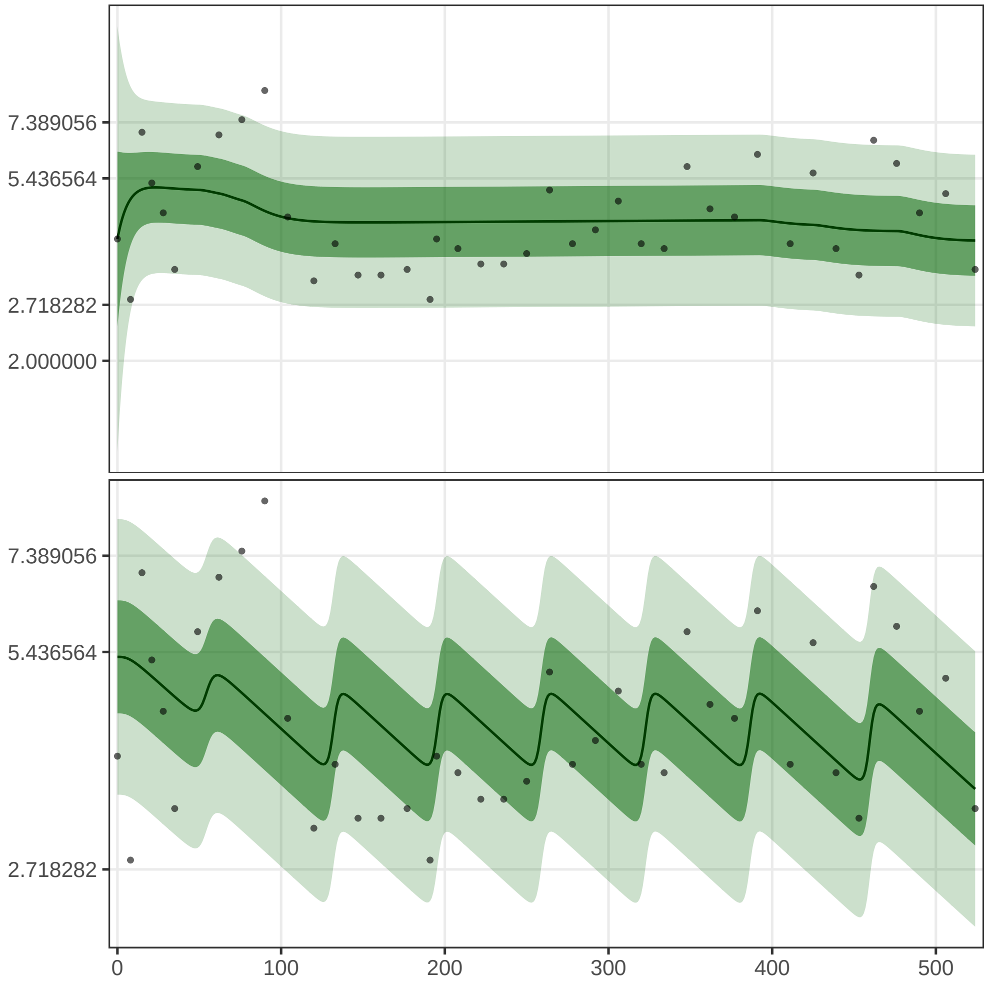


Supplementary Figure 22. The models TCM (top) and JM (bottom) fit to the full dataset of patient 21 with time in days on the x-axis and leukocyte count on the y-axis. The fitted mean is the black line and probability intervals (50%, 90%) are plotted in green.

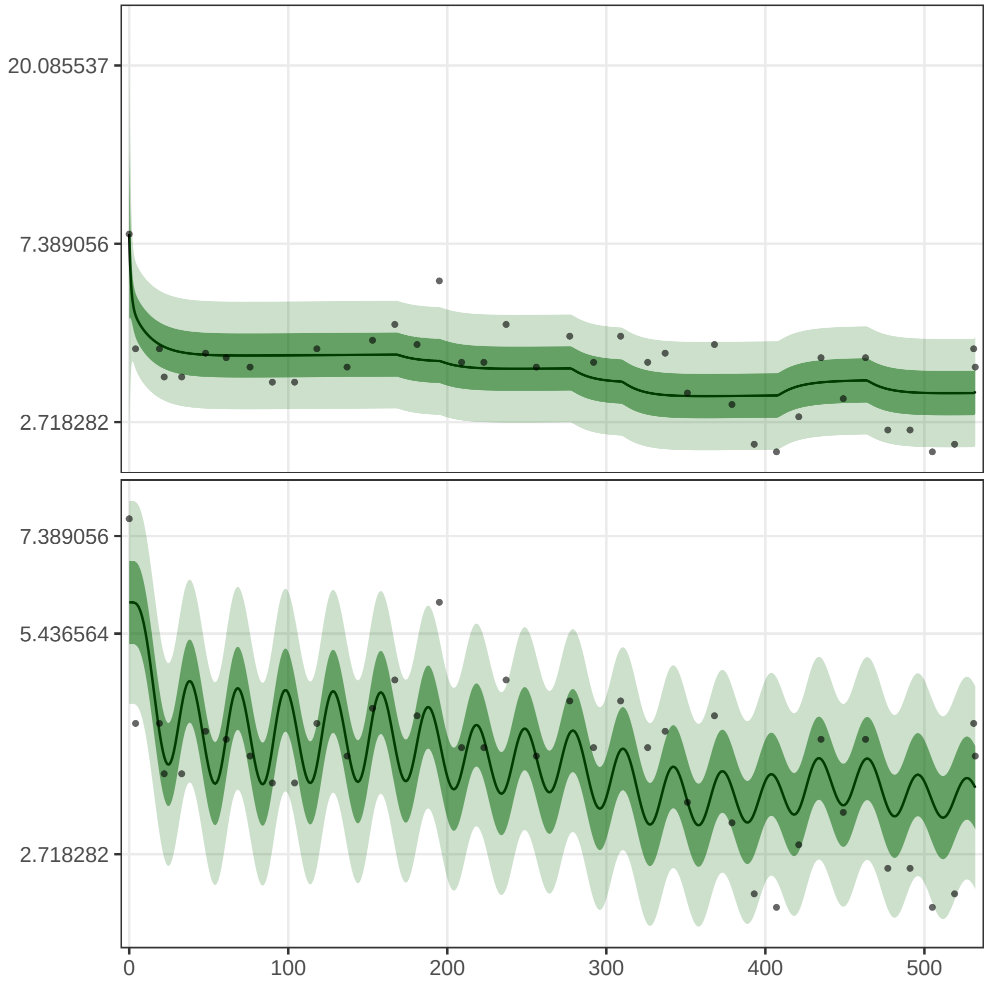


Supplementary Figure 23. The models TCM (top) and JM (bottom) fit to the full dataset of patient 22 with time in days on the x-axis and leukocyte count on the y-axis. The fitted mean is the black line and probability intervals (50%, 90%) are plotted in green.

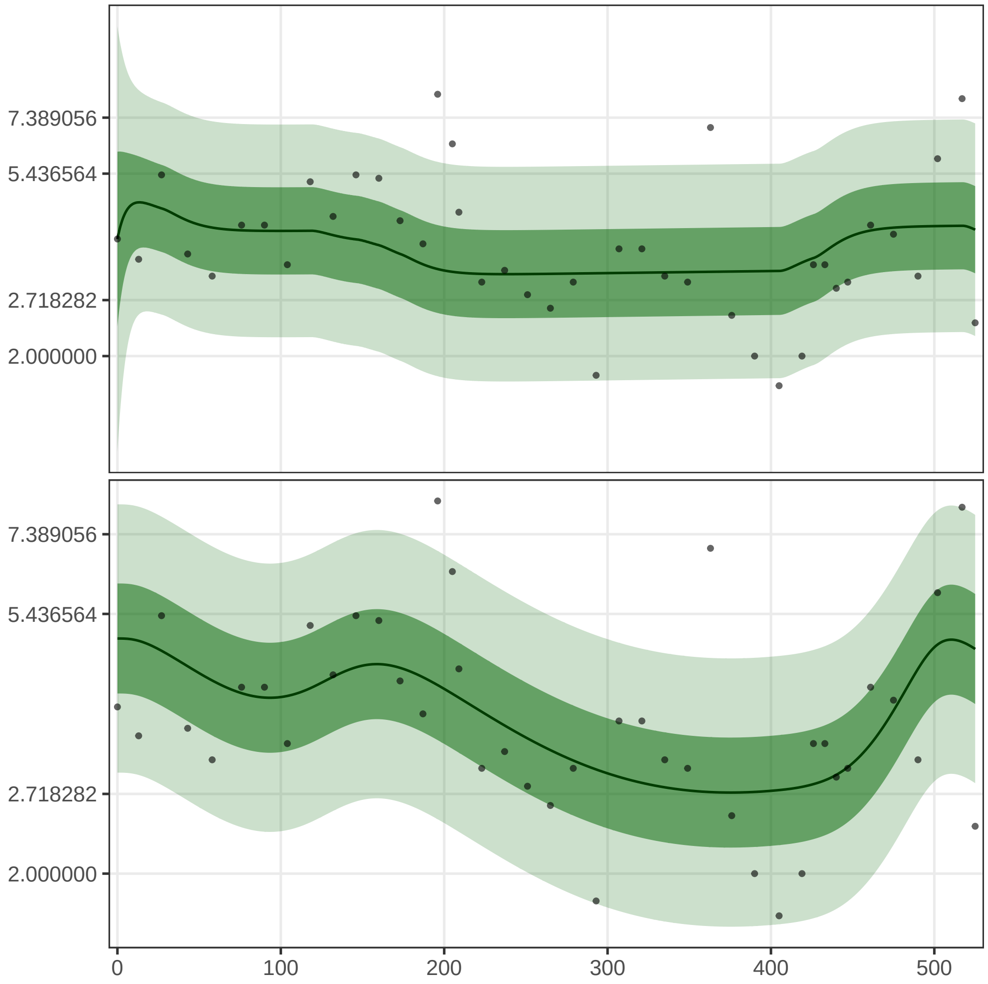


Supplementary Figure 24. The models TCM (top) and JM (bottom) fit to the full dataset of patient 23 with time in days on the x-axis and leukocyte count on the y-axis. The fitted mean is the black line and probability intervals (50%, 90%) are plotted in green.

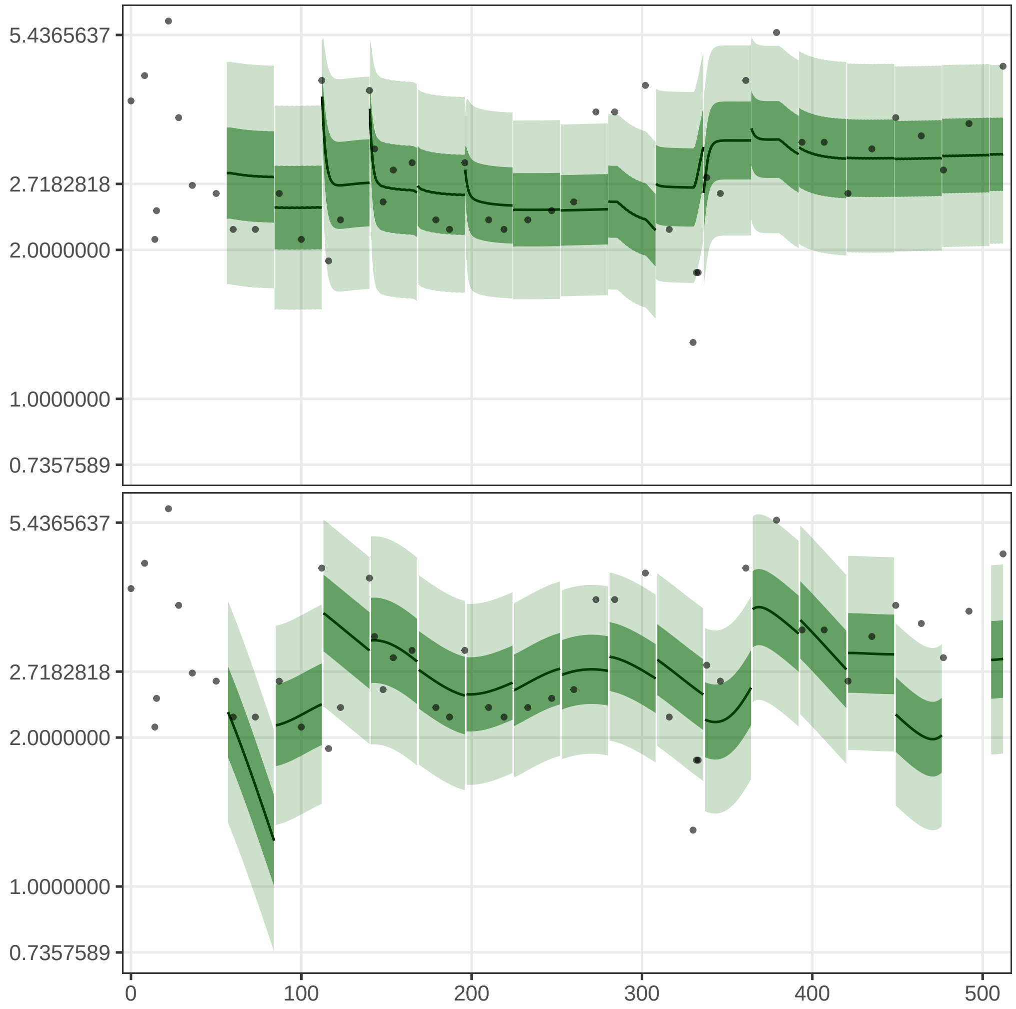


Supplementary Figure 25. Predictions for patient 1 at each round of time series cross-validation with the four week prediction horizon for the models TCM (top) and JM (bottom).

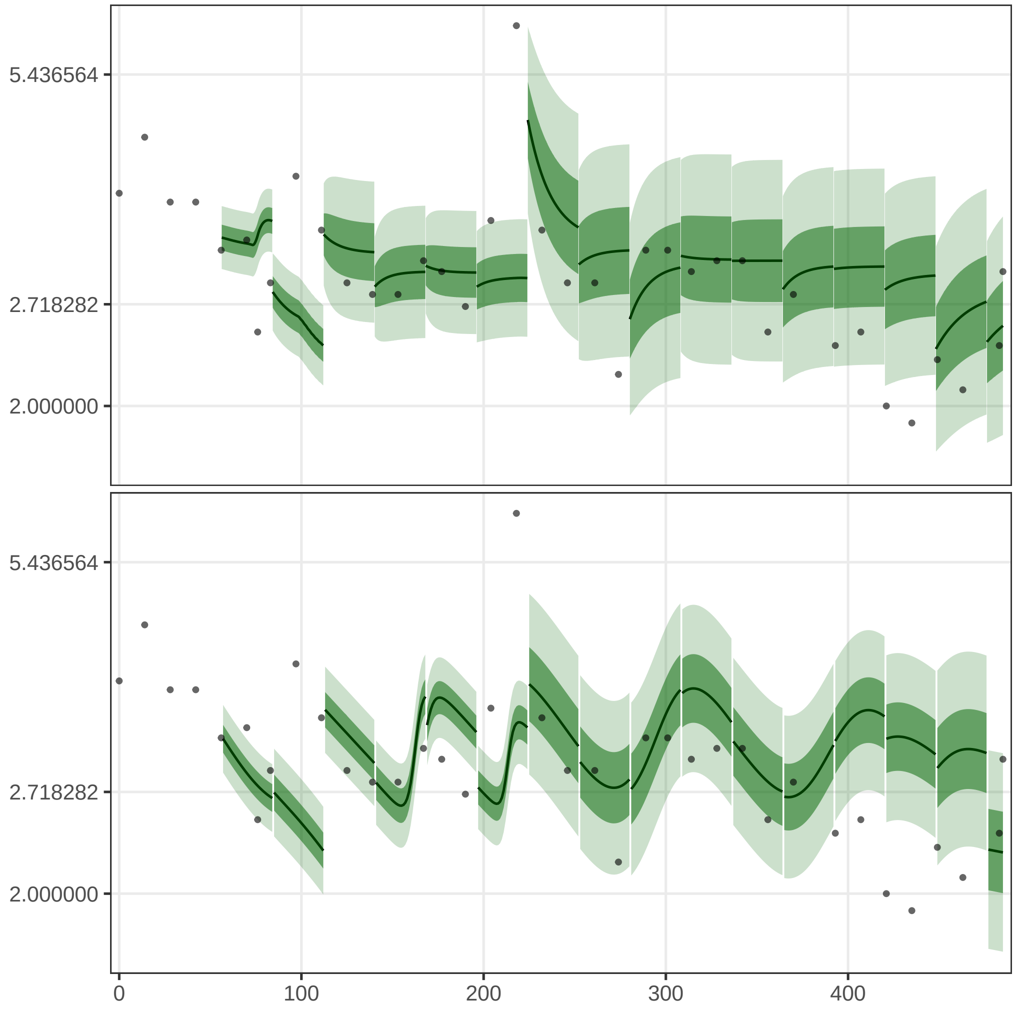


Supplementary Figure 26. Predictions for patient 2 at each round of time series cross-validation with the four week prediction horizon for the models TCM (top) and JM (bottom).

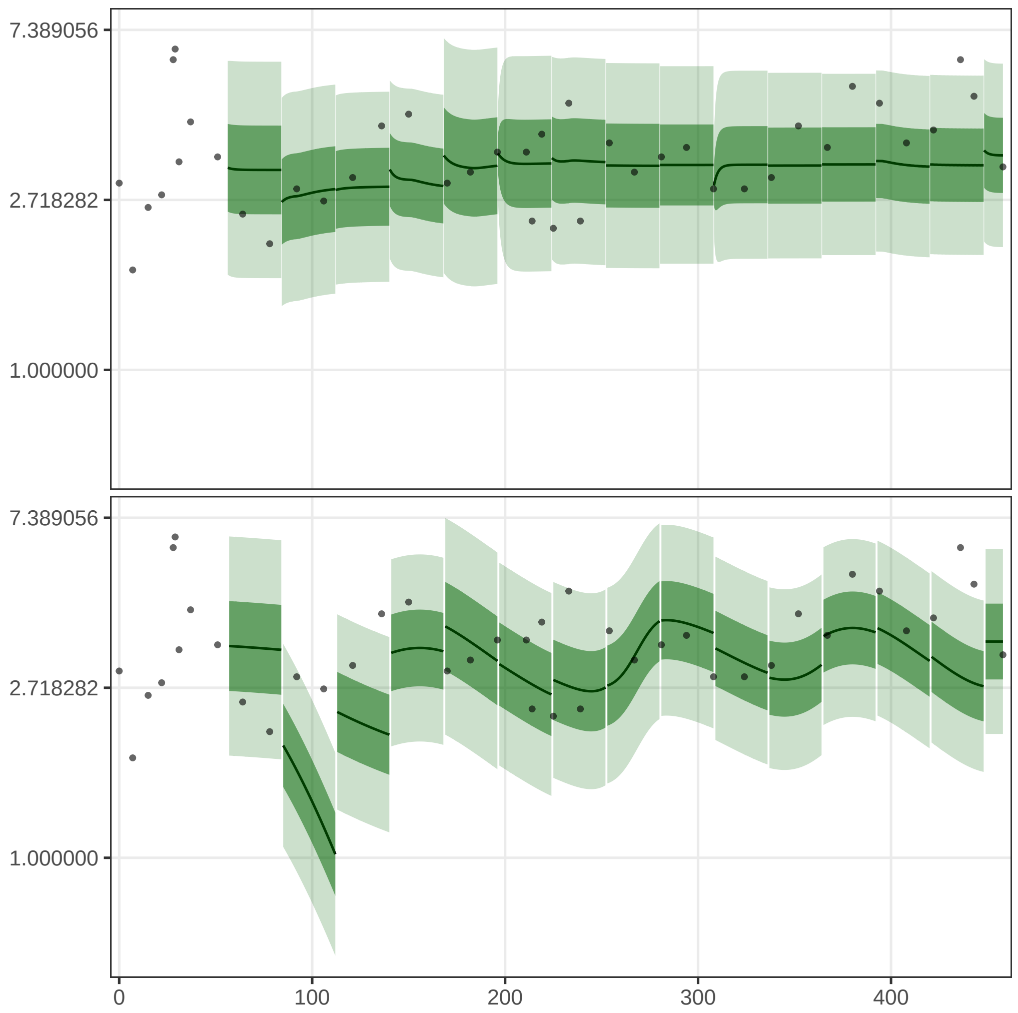


Supplementary Figure 27. Predictions for patient 3 at each round of time series cross-validation with the four week prediction horizon for the models TCM (top) and JM (bottom).

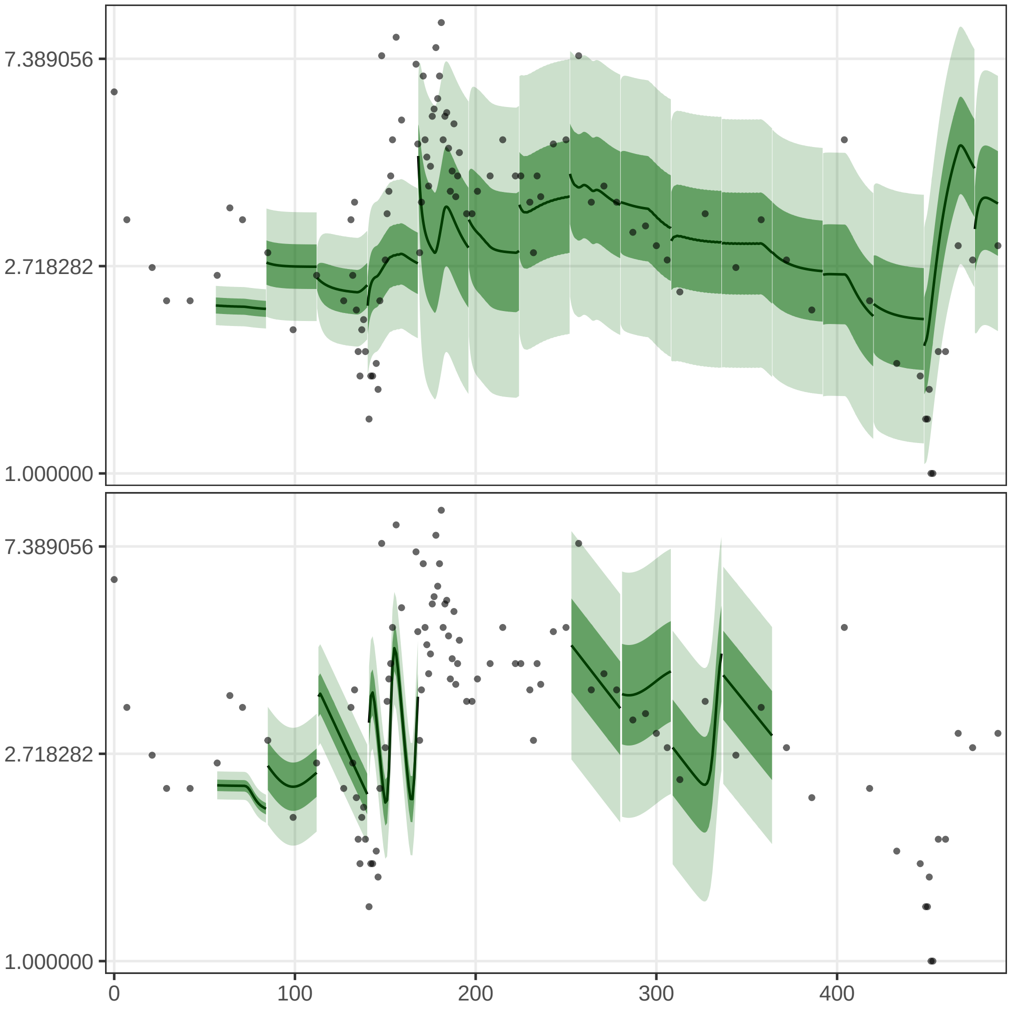


Supplementary Figure 28. Predictions for patient 4 at each round of time series cross-validation with the four week prediction horizon for the models TCM (top) and JM (bottom).

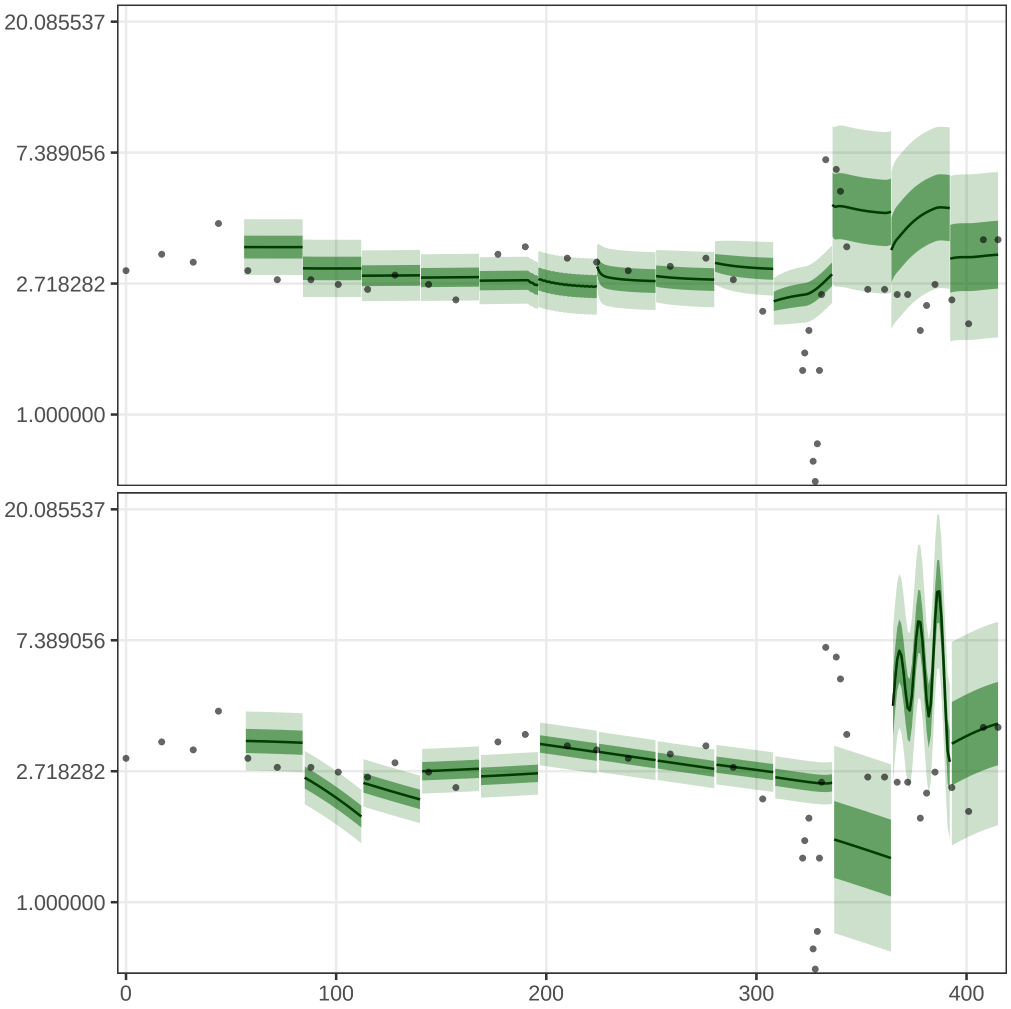


Supplementary Figure 29. Predictions for patient 5 at each round of time series cross-validation with the four week prediction horizon for the models TCM (top) and JM (bottom).

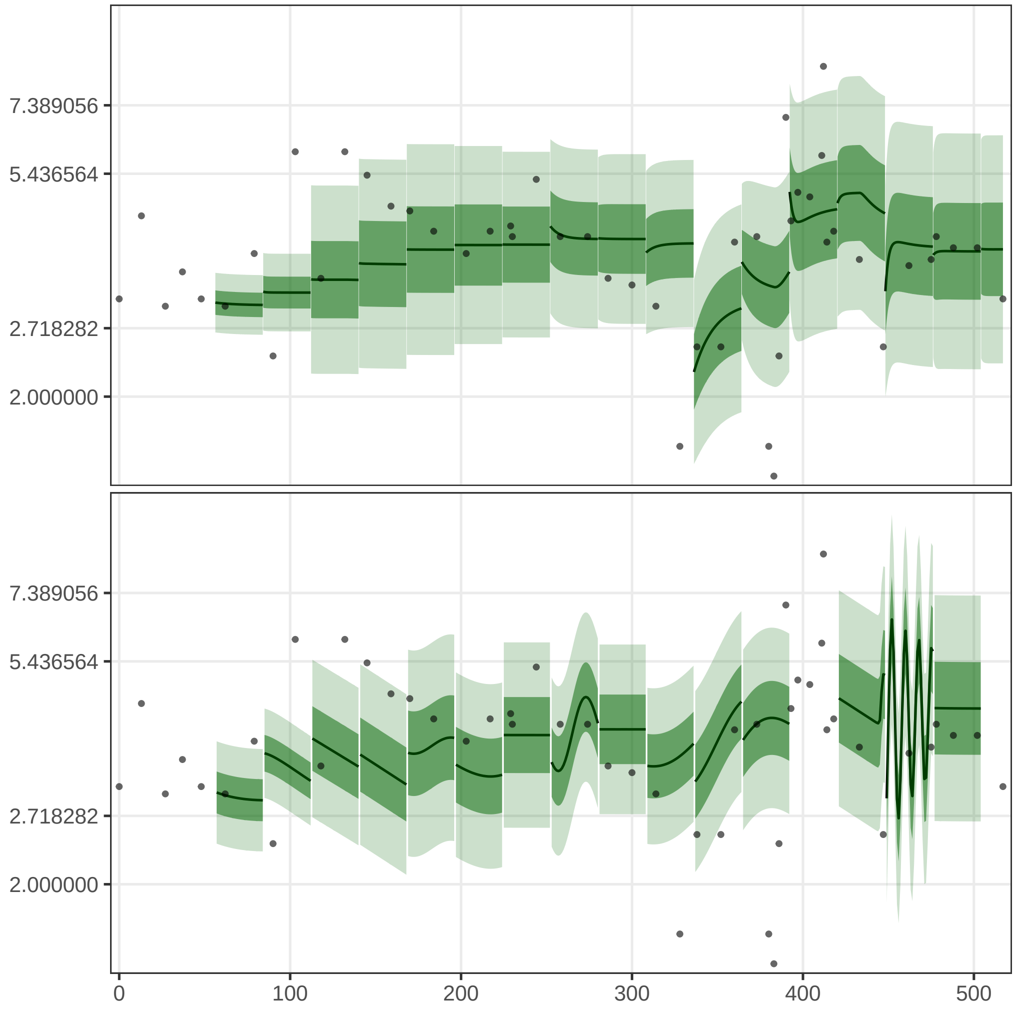


Supplementary Figure 30. Predictions for patient 6 at each round of time series cross-validation with the four week prediction horizon for the models TCM (top) and JM (bottom).

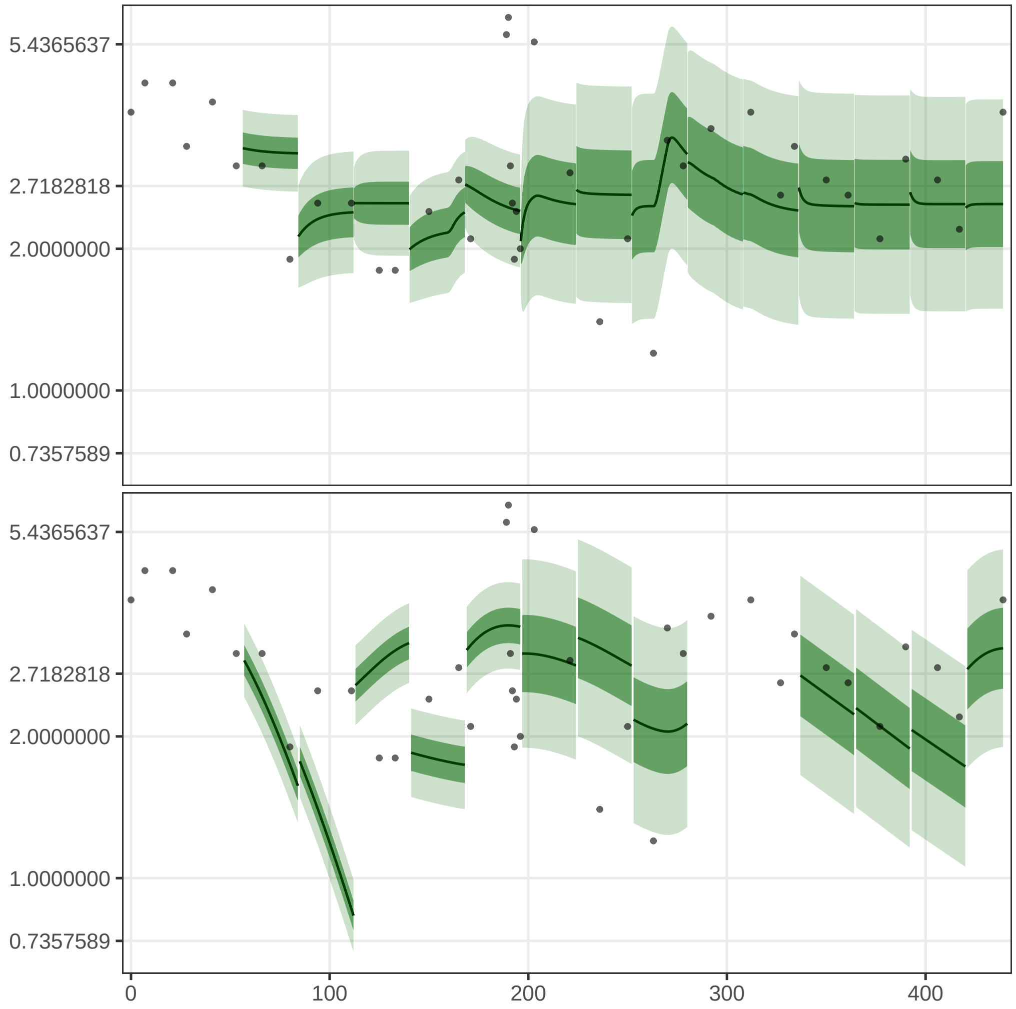


SUPPLEMENTARY FIGURE 31. Predictions for patient 7 at each round of time series cross-validation with the four week prediction horizon for the models TCM (top) and JM (bottom).

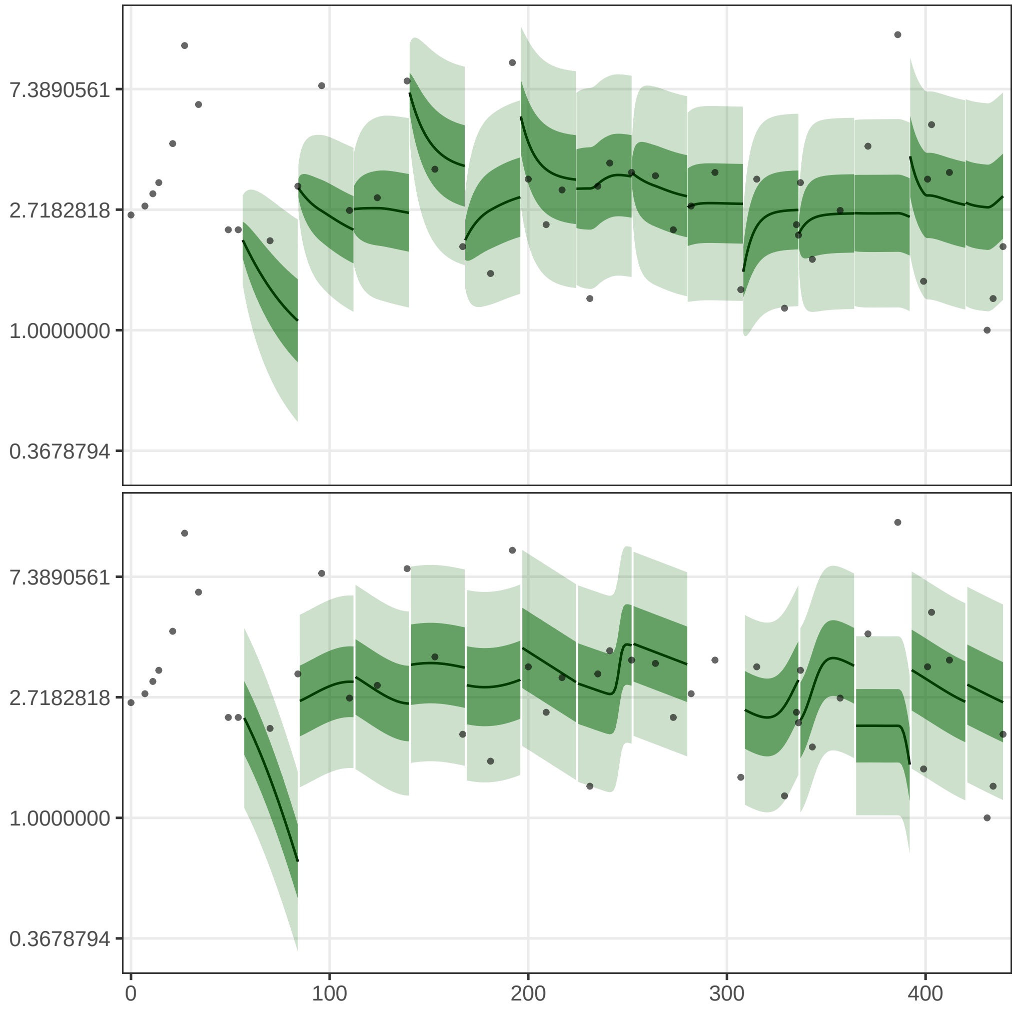


Supplementary Figure 32. Predictions for patient 8 at each round of time series cross-validation with the four week prediction horizon for the models TCM (top) and JM (bottom).

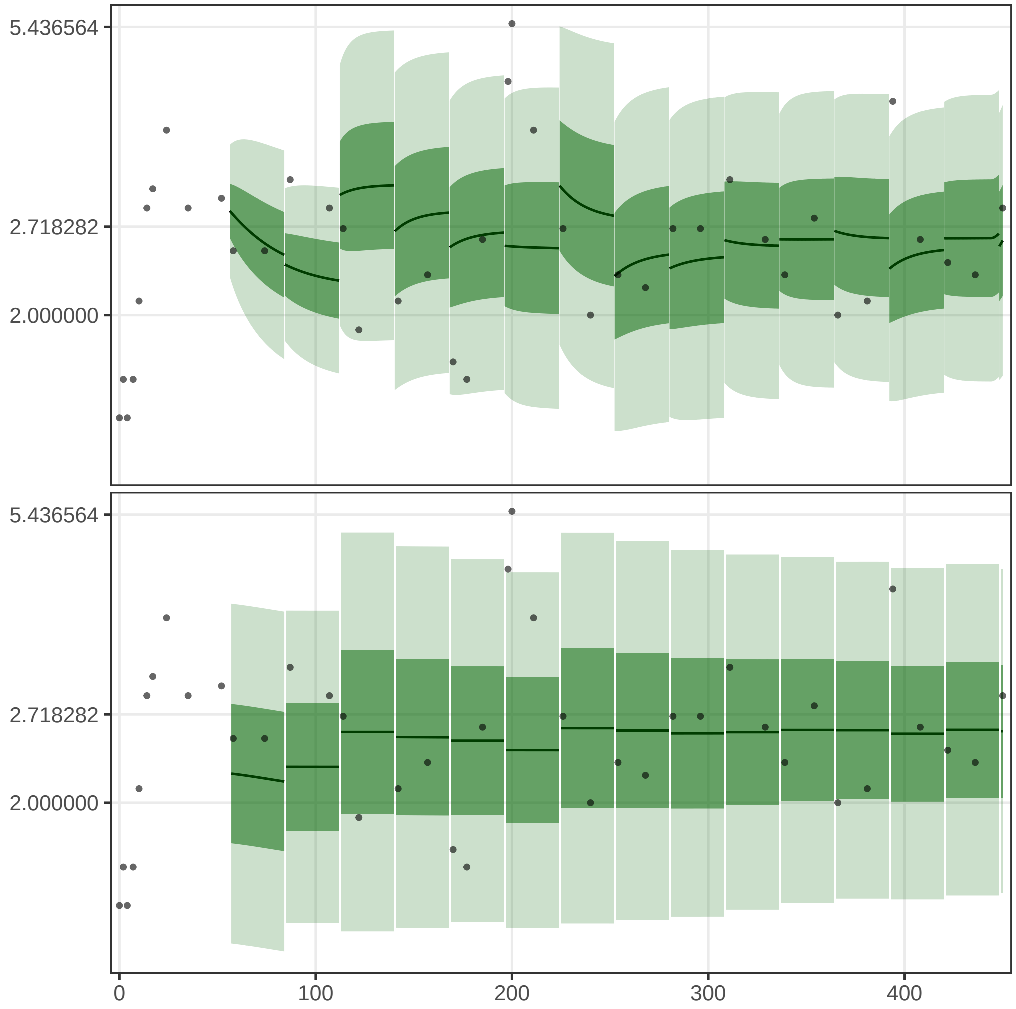


SUPPLEMENTARY FIGURE 33. Predictions for patient 9 at each round of time series cross-validation with the four week prediction horizon for the models TCM (top) and JM (bottom).

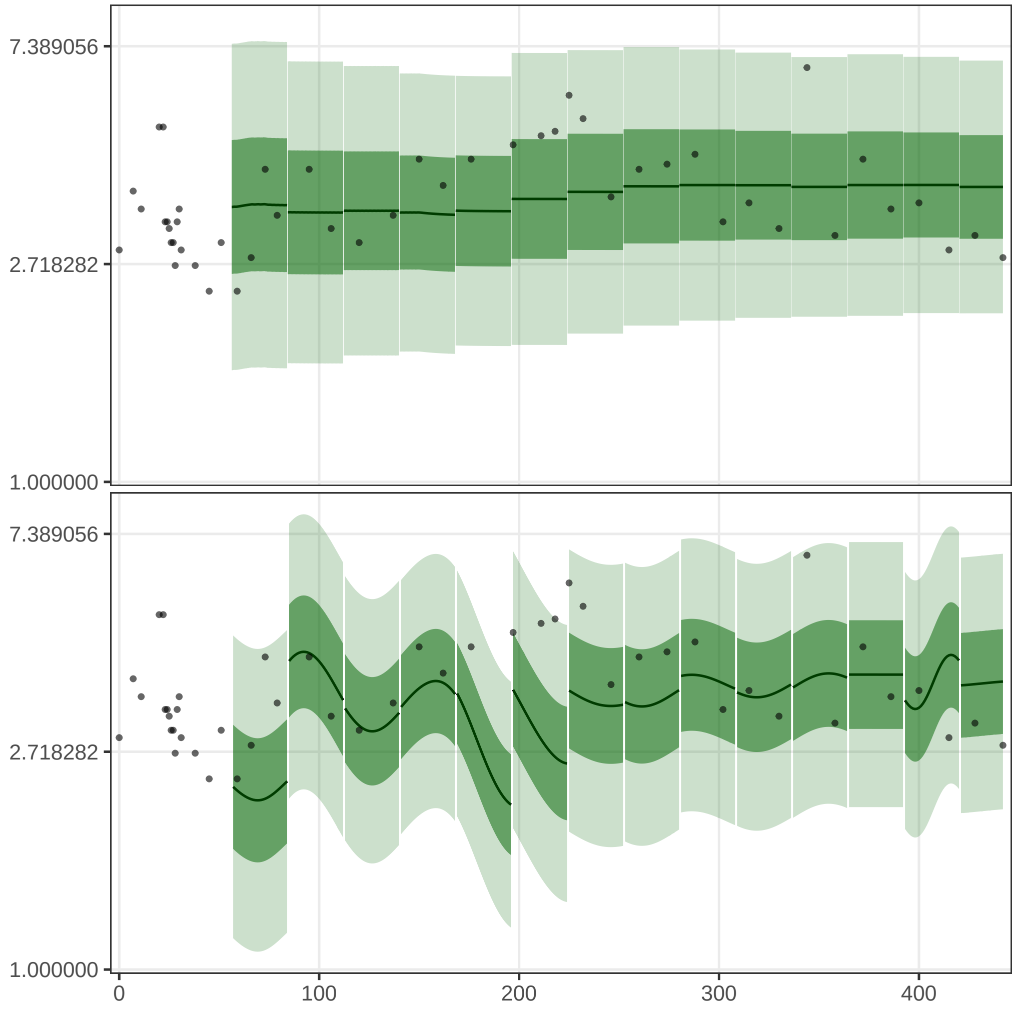


Supplementary Figure 34. Predictions for patient 10 at each round of time series cross-validation with the four week prediction horizon for the models TCM (top) and JM (bottom).

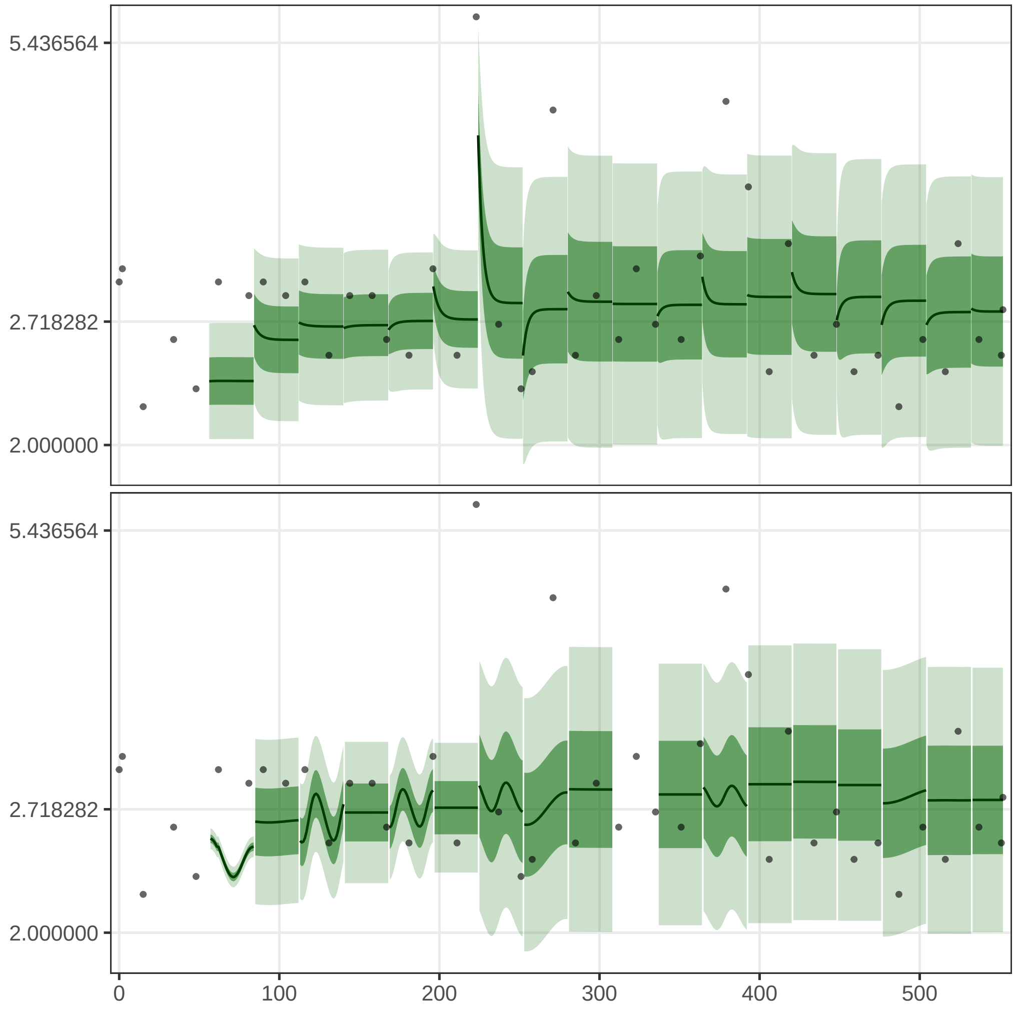


Supplementary Figure 35. Predictions for patient 11 at each round of time series cross-validation with the four week prediction horizon for the models TCM (top) and JM (bottom).

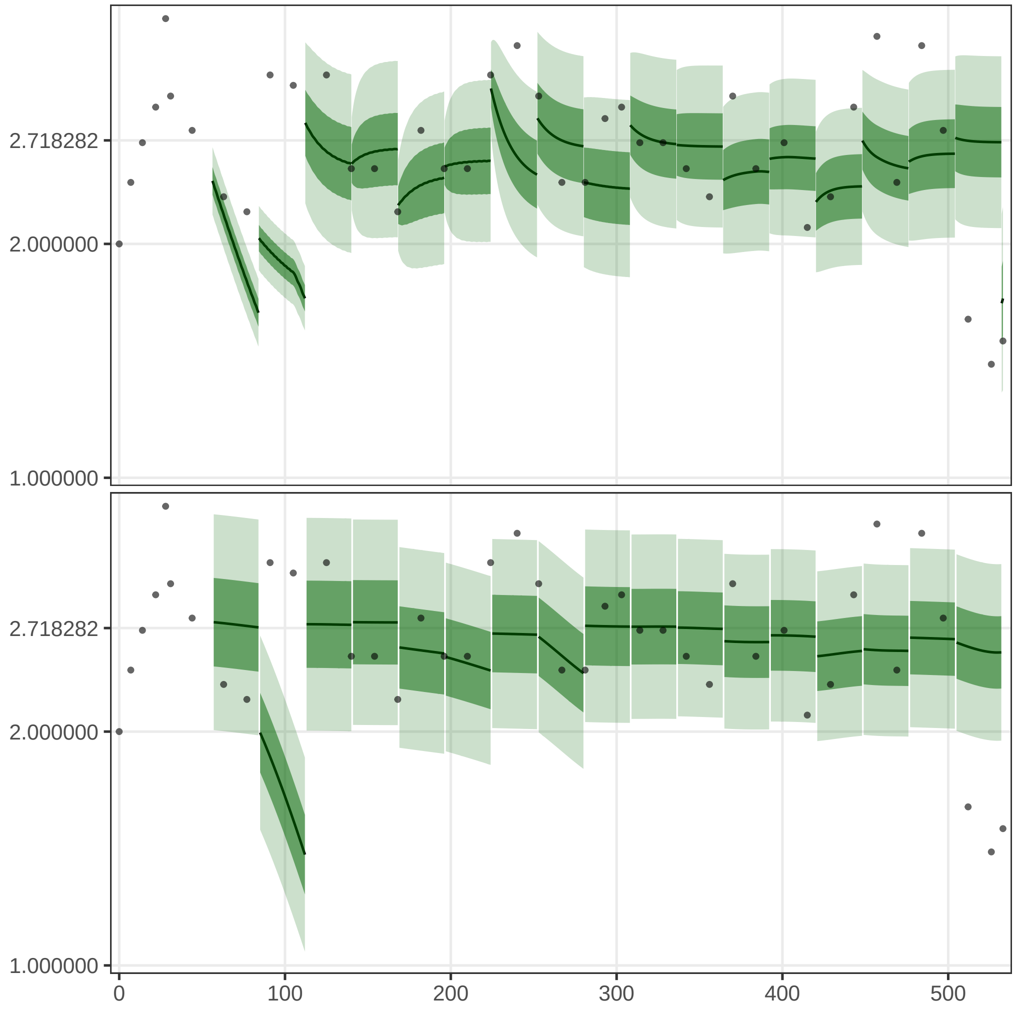


Supplementary Figure 36. Predictions for patient 12 at each round of time series cross-validation with the four week prediction horizon for the models TCM (top) and JM (bottom).

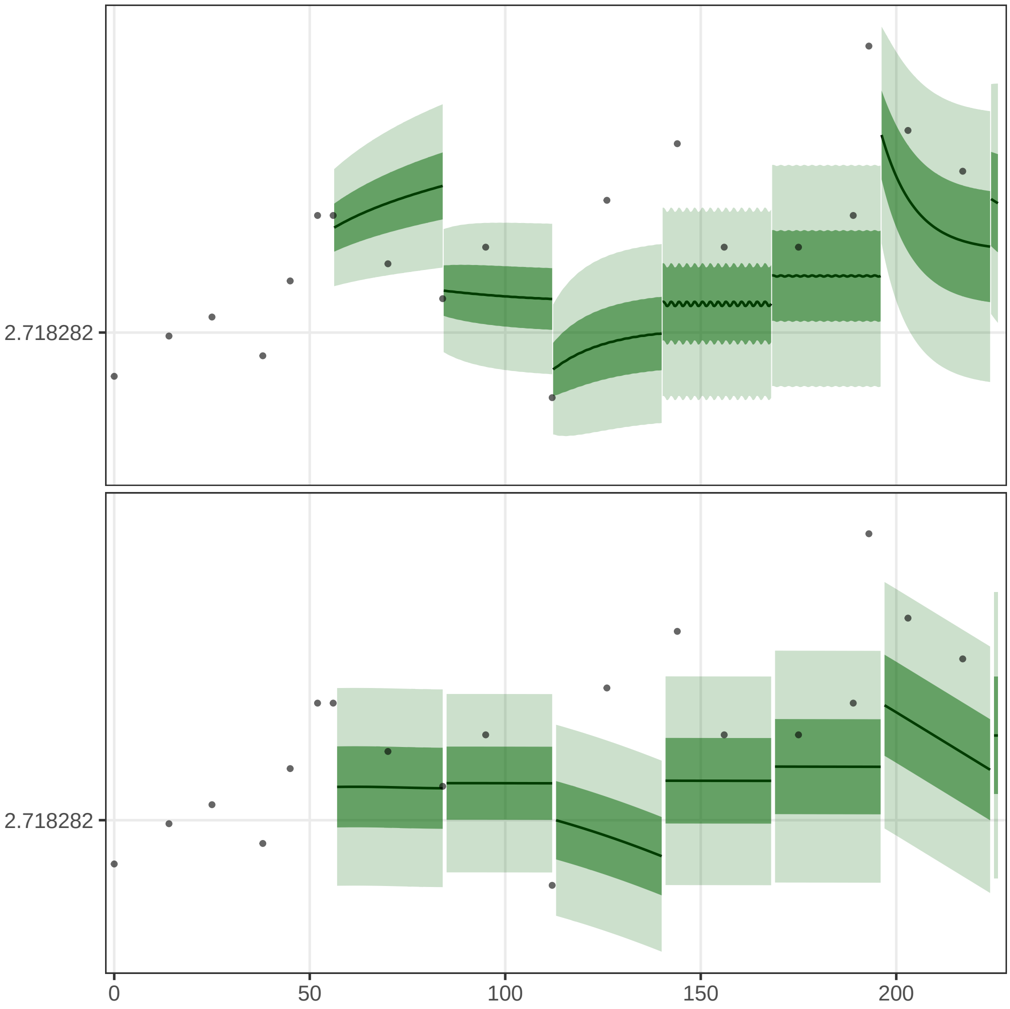


Supplementary Figure 37. Predictions for patient 13 at each round of time series cross-validation with the four week prediction horizon for the models TCM (top) and JM (bottom).

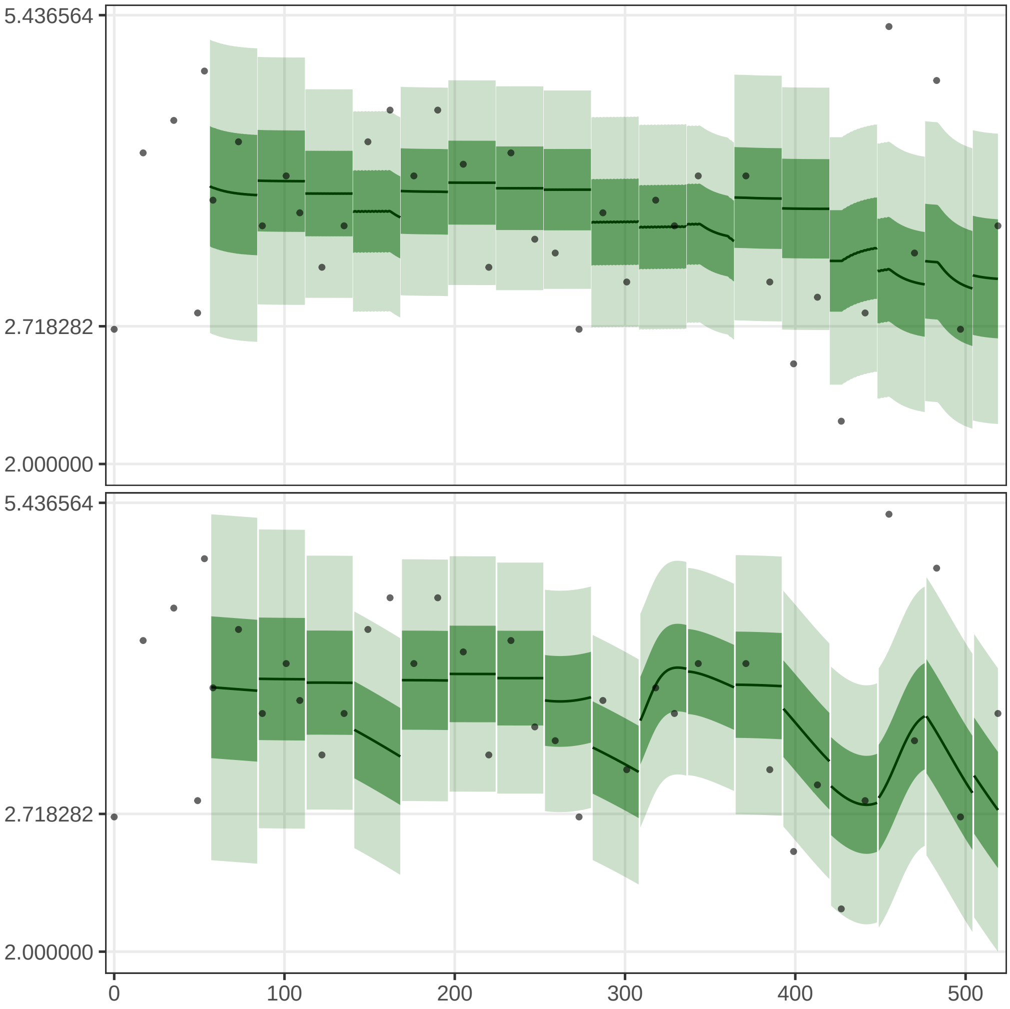


Supplementary Figure 38. Predictions for patient 14 at each round of time series cross-validation with the four week prediction horizon for the models TCM (top) and JM (bottom).

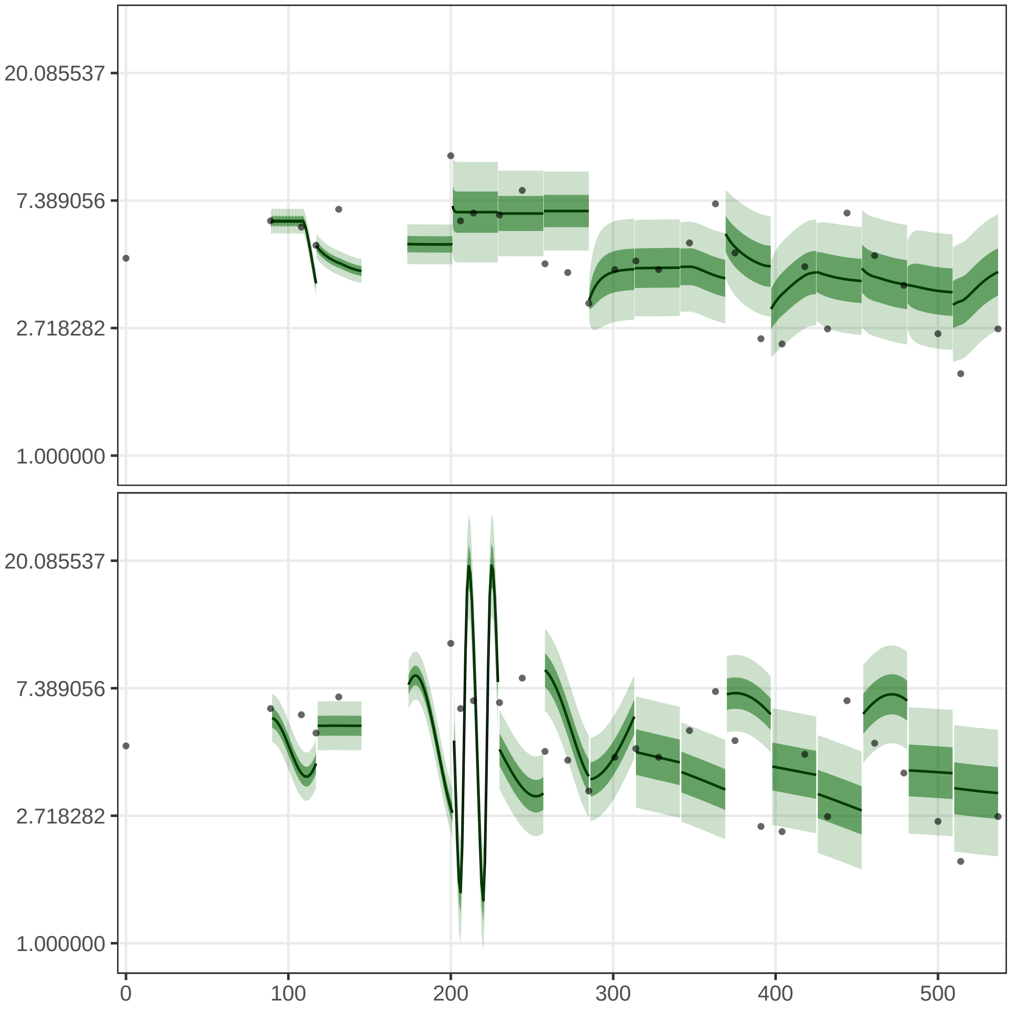


Supplementary Figure 39. Predictions for patient 15 at each round of time series cross-validation with the four week prediction horizon for the models TCM (top) and JM (bottom).

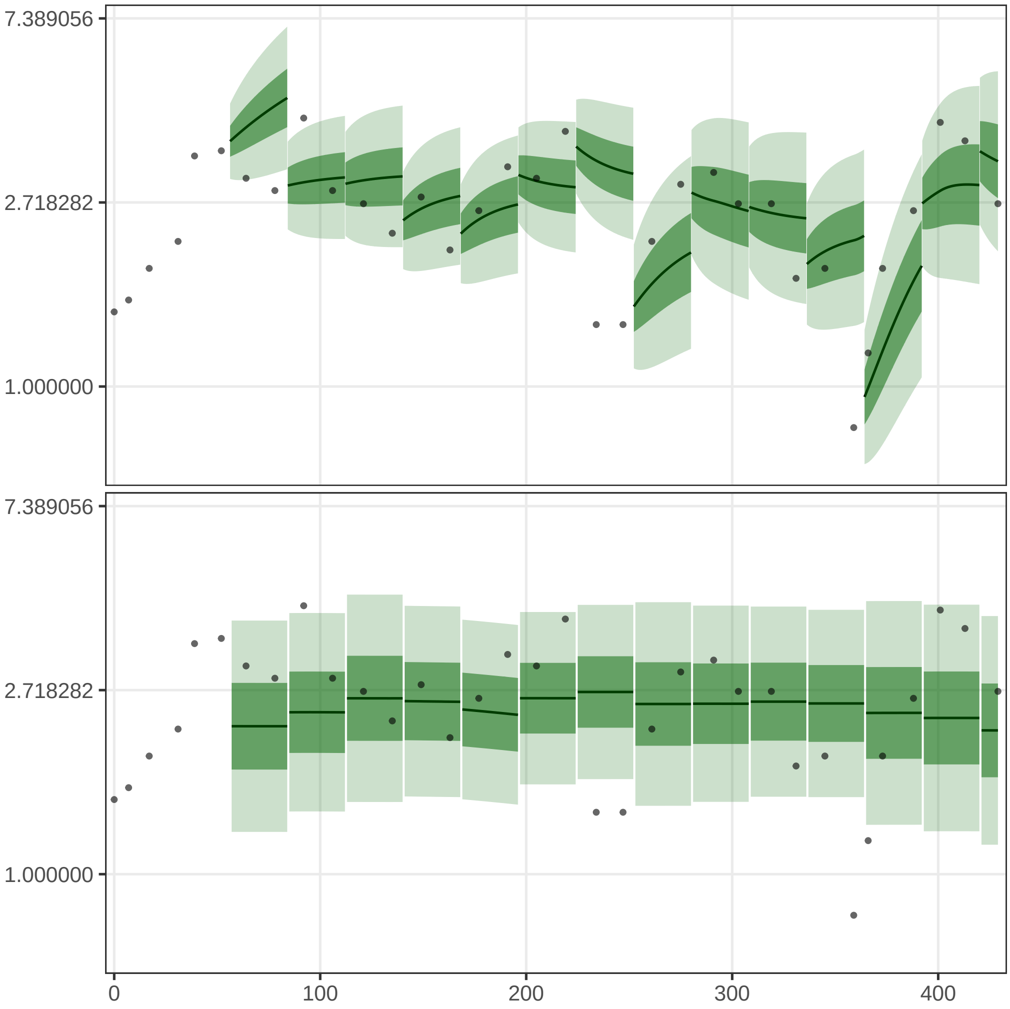


Supplementary Figure 40. Predictions for patient 16 at each round of time series cross-validation with the four week prediction horizon for the models TCM (top) and JM (bottom).

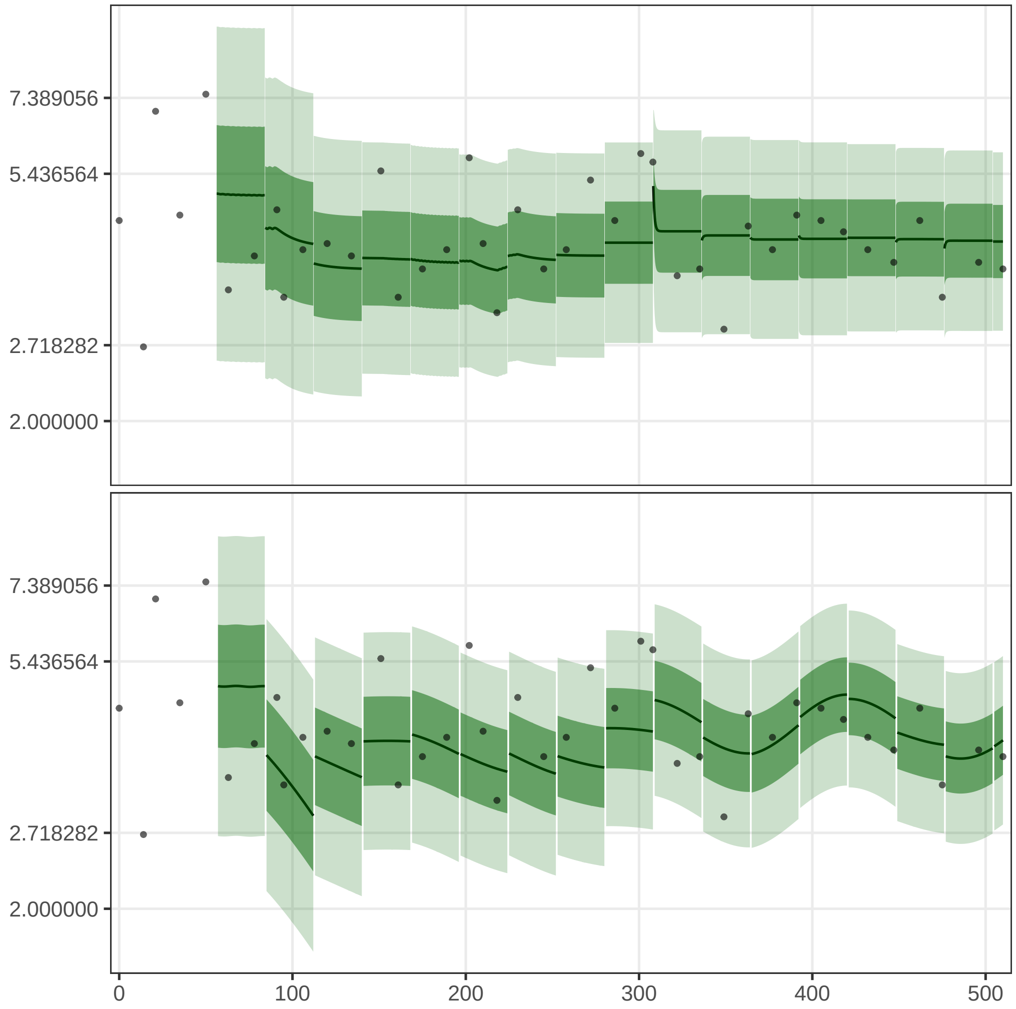


Supplementary Figure 41. Predictions for patient 17 at each round of time series cross-validation with the four week prediction horizon for the models TCM (top) and JM (bottom).

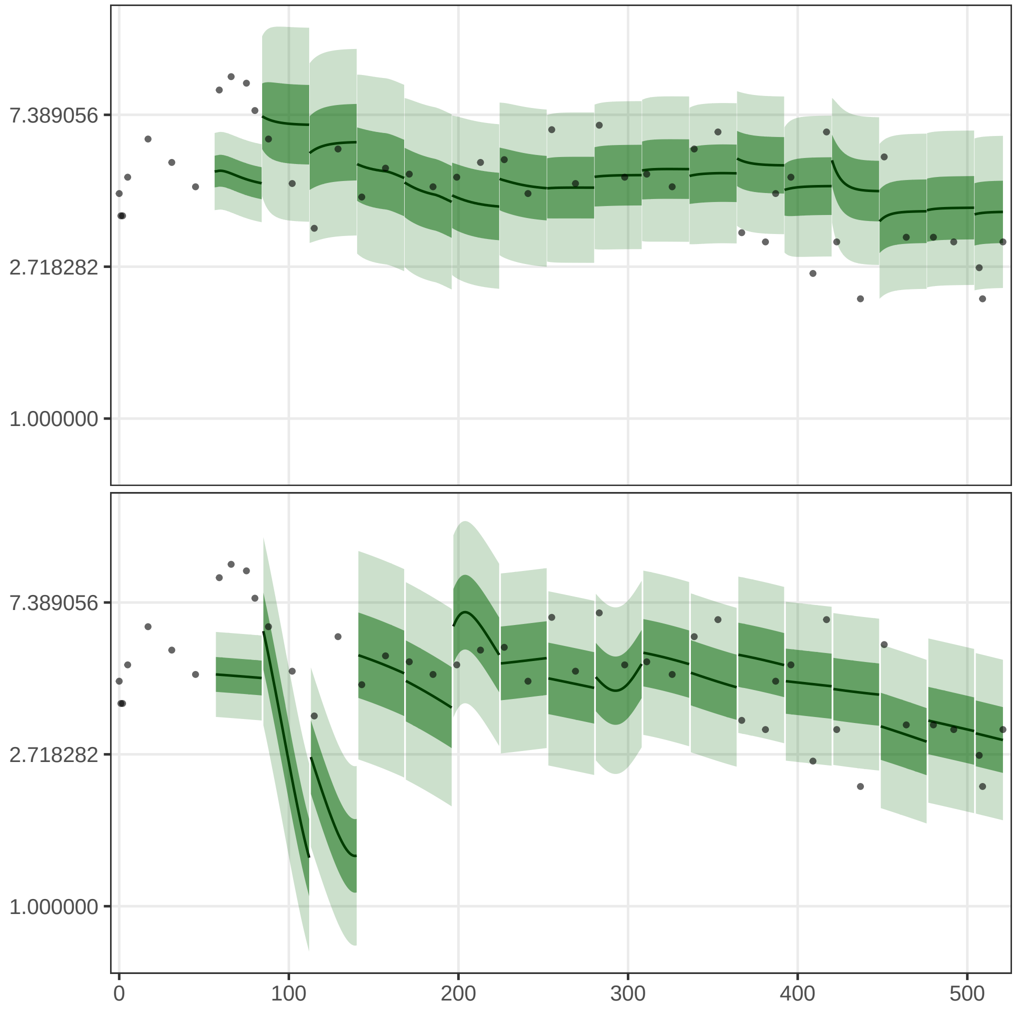


Supplementary Figure 42. Predictions for patient 18 at each round of time series cross-validation with the four week prediction horizon for the models TCM (top) and JM (bottom).

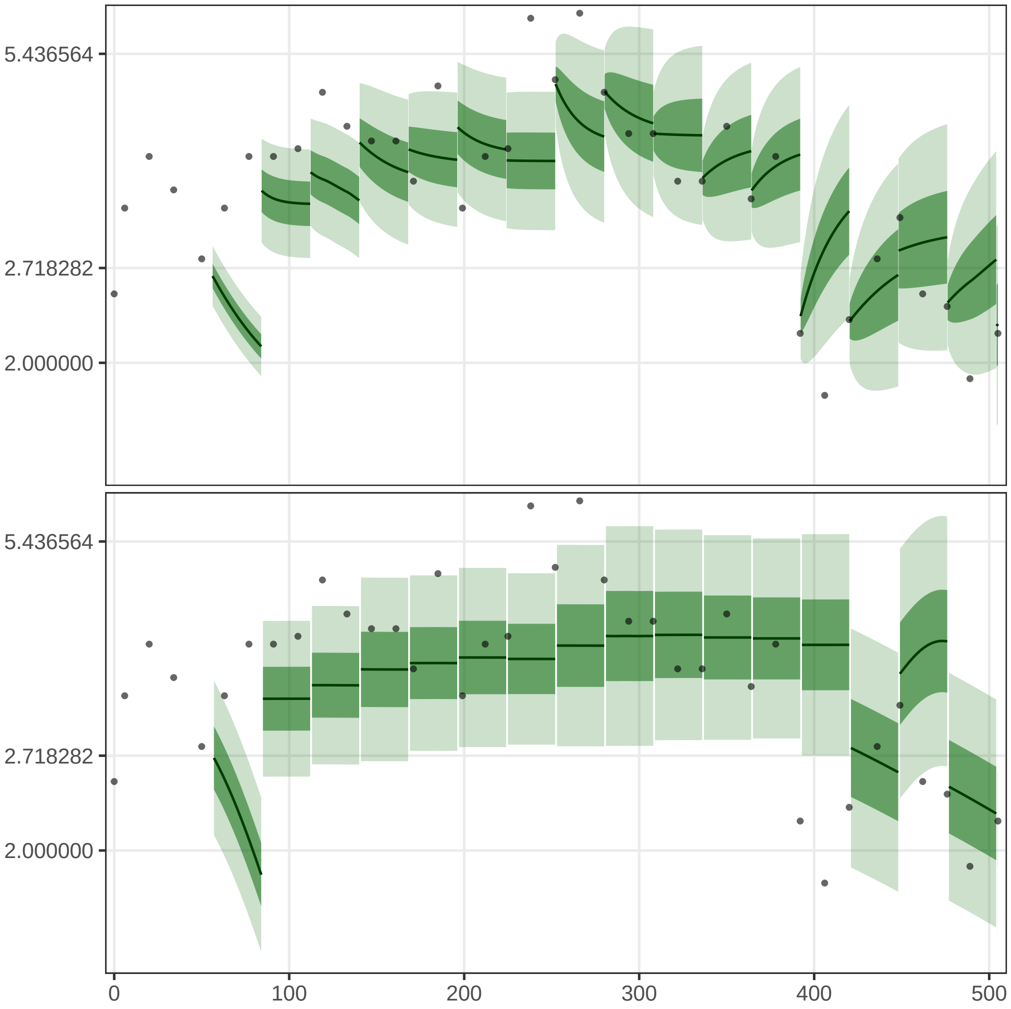


Supplementary Figure 43. Predictions for patient 19 at each round of time series cross-validation with the four week prediction horizon for the models TCM (top) and JM (bottom).

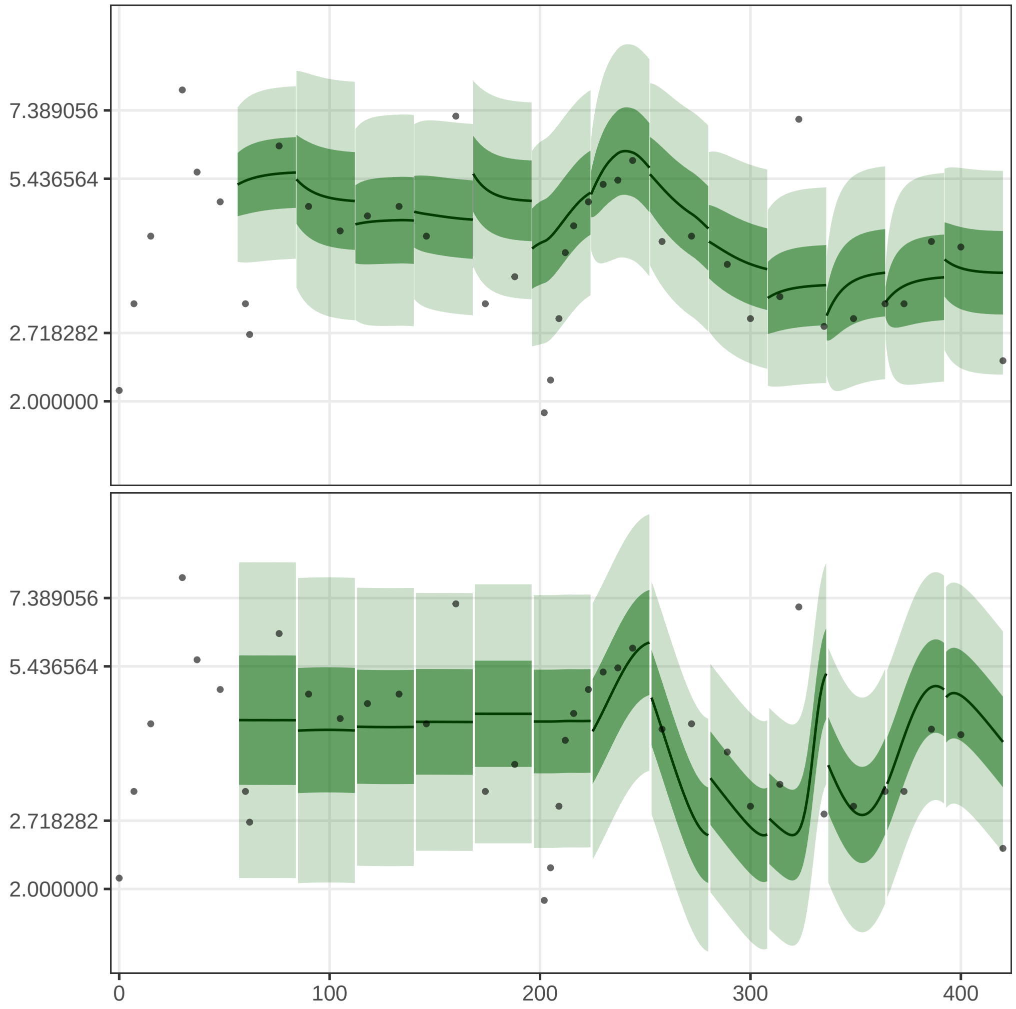


Supplementary Figure 44. Predictions for patient 20 at each round of time series cross-validation with the four week prediction horizon for the models TCM (top) and JM (bottom).

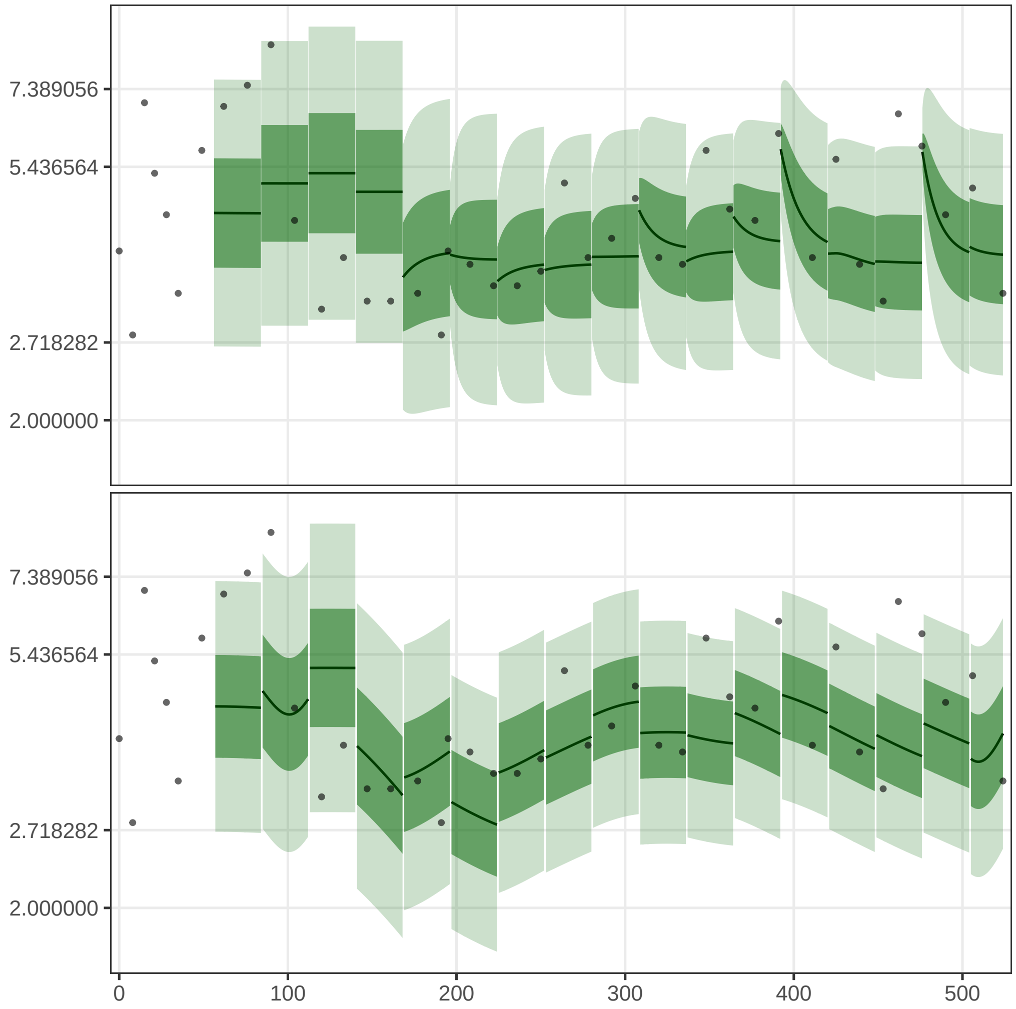


Supplementary Figure 45. Predictions for patient 21 at each round of time series cross-validation with the four week prediction horizon for the models TCM (top) and JM (bottom).

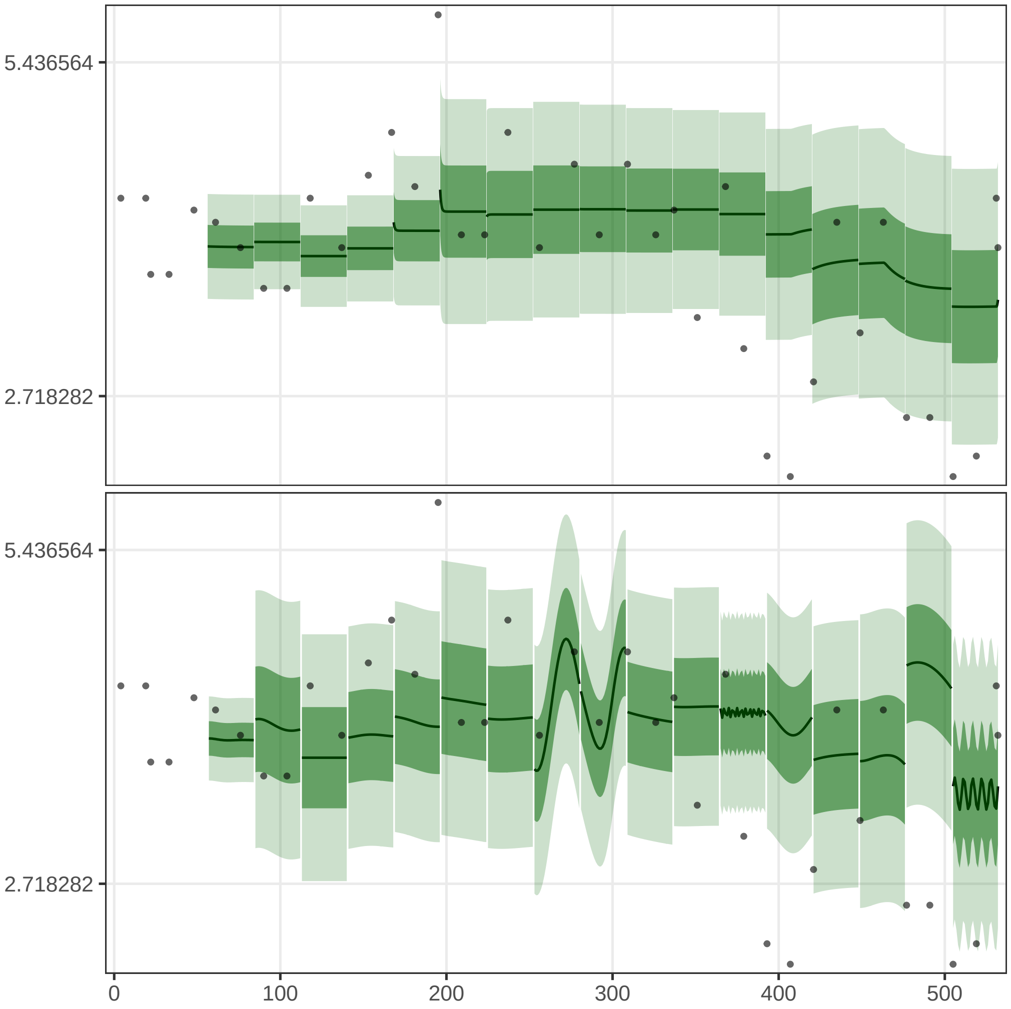


Supplementary Figure 46. Predictions for patient 22 at each round of time series cross-validation with the four week prediction horizon for the models TCM (top) and JM (bottom).

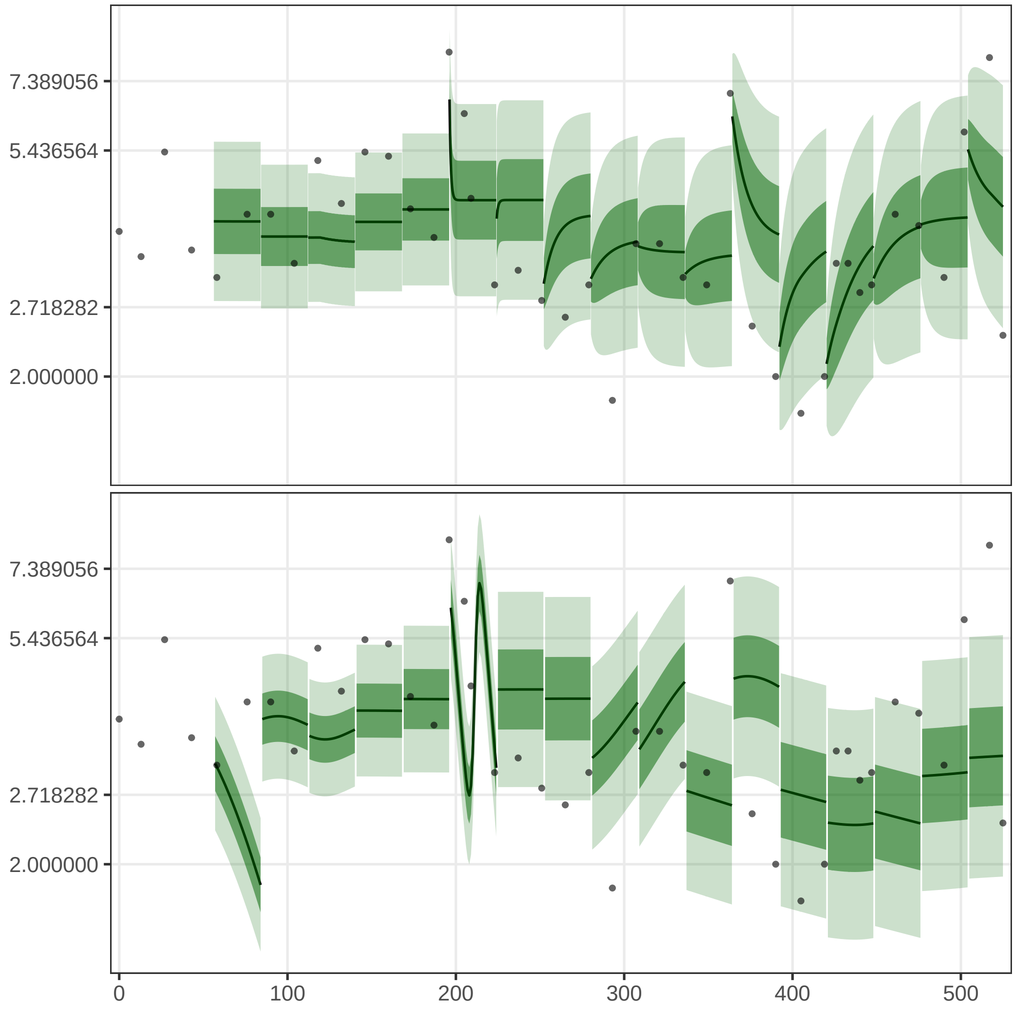


Supplementary Figure 47. Predictions for patient 23 at each round of time series cross-validation with the four week prediction horizon for the models TCM (top) and JM (bottom).

## References

[1] Devaraj Jayachandran, Ann E Rundell, Robert E Hannemann, Terry A Vik, and Doraiswami Ramkrishna. Optimal chemotherapy for leukemia: a model-based strategy for individualized treatment. *PloS one*, 9(10): e109623, 2014.

[2] Devaraj Jayachandran, José Laínez-Aguirre, Ann Rundell, Terry Vik, Robert Hannemann, Gintaras Reklaitis, and Doraiswami Ramkrishna. Model-based individualized treatment of chemotherapeutics: Bayesian population modeling and dose optimization. *PloS one*, 10(7):e0133244, 2015.

[3] Erik K Amundsen, Petter Urdal, Tor-Arne Hagve, Mette R Holthe, and Carola E Henriksson. Absolute neutrophil counts from automated hematology instruments are accurate and precise even at very low levels. *American journal of clinical pathology*, 137(6):862–869, 2012.
